# Environmental Variables Influence on Spatial Distribution of Bacterial Communities Across the English Channel in Two Main Productive Seasons

**DOI:** 10.1111/1758-2229.70213

**Published:** 2025-10-12

**Authors:** Luen‐Luen Li, Nicolas Delatre, Zéline Hubert, Luis Felipe Artigas, Sébastien Monchy

**Affiliations:** ^1^ Université du Littoral Côte d'Opale, CNRS, Univ. Lille, UMR 8187, LOG, Laboratoire d'Océanologie et de Géosciences Wimereux France

**Keywords:** bacterial community structure, environmental variables, high throughput sequencing, the English Channel

## Abstract

The English Channel features a wide range of ecological habitats that host numerous biodiversity resources and are submitted to natural and/or anthropogenic pressures. During the ‘EcoPel’ oceanographic campaigns (spring and summer 2018) in French waters of the English Channel and North Sea, a variety of coastal pelagic habitats were sampled for analysing environmental variables and bacterial communities. Results of PCA suggest that main environmental variables were SPM, POM, PIM, salinity and NO_2_^−^/NO_3_^+^ in spring and salinity, SPM, Si and Chl‐*a* in summer. The Shannon index suggested summer alpha diversity had higher richness and equitability compared to spring. A clear seasonality in the bacterial community structure was also revealed by hierarchical cluster analysis. Most of the spring communities had a higher proportion of Bacteroidetes while most of the summer communities had a higher proportion of Proteobacteria, Actinobacteria, Verrucomicrobia and Planctomycetes. Based on distance correlations and statistical significance, the spring taxonomic composition was correlated with Chl‐*a*, PO_4_, POM, SPM and PIM, while the summer composition was correlated with salinity. According to hierarchical cluster analyses, both environmental variables and bacterial communities seem to be clustered in parallel with the coast, evidencing the main influence of coastal‐offshore gradients and implying possible links with river inputs and phytoplankton/algae dynamics.

## Graphical abstract

In the English Channel, the main environmental variables were SPM, POM, PIM, NO_2_^−^/NO_3_^+^ and salinity in spring and salinity, SPM, Si and Chl‐*a* in summer. Most of the spring communities had a higher proportion of Bacteroidetes and Proteobacteria, while most of the summer communities had a higher proportion of Proteobacteria, Bacteroidetes, Actinobacteria, Cyanobacteria and Verrucomicrobia. The summer alpha diversity had higher richness and equitability compared to spring.

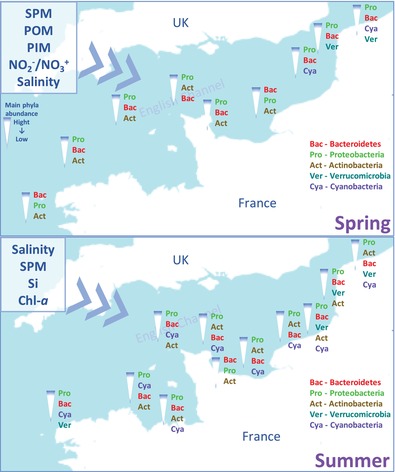

## 1 Introduction

In marine ecosystems, microorganisms play crucial roles in ecosystem function (primary productivity, re‐mineralization of nutrients, microbial loop, etc.) and resilience (Azam et al. 1983; Buchan et al. 2014). They not only contribute to biogeochemical fluxes, biomass production and fishery yields by controlling nutrient cycling at the base of food webs, but also influence the atmosphere and climate through CO_2_ fixation/O_2_ production, greenhouse gases sequestration and climate gases emission (Buchan et al. 2014; Yoch 2002). The community structure of microbiota remains in a dynamic balance continuously in response to biotic and abiotic parameters such as nutrients, physical and chemical conditions. Growth, immigration and physical concentration directly affect the abundance of taxa, whereas grazing, lysis, sedimentation, dilution and emigration generally act to stabilize population fluctuations (Moreira and López‐García 2002; James 2005; Normand et al. 2015). Consequently, although species are competing for resources, they also coexist in water masses having apparently similar properties. However, many details of microbiota's seasonal and spatial dynamics, especially at molecular and genetic levels, are neither well characterized nor understood.

The English Channel (EC) is a transitional marginal sea between the warm waters of the Atlantic Ocean on the West and the cold waters of the North Sea on the East, and is characterised by strong tidal currents that generate high turbulence (Idier et al. 2012). Tidal currents and eastward winds are responsible for quasi‐homogeneous vertical mixing and influence water mass transports from the Atlantic Ocean to the North Sea (average transit time 6–12 months) (Pingree and Griffiths 1978; du Bois and Dumas 2005; Salomon and Breton 1993). In addition, river inputs (such as the Seine, the Somme, etc.) influence water circulation as well as salt and nutrient concentration in the Channel. The English Channel is separated into three distinct areas: Western English Channel, Central English Channel‐Bay of Seine and Eastern English Channel (Figure S1). Atlantic waters greatly influence the western area. The entrance of the English Channel is marked by the seasonal Ushant tidal front, with stratified water masses on the West and mixed water masses on the East, acting as an ecological boundary for microbial plankton communities (Group, G 1988; Lemonnier et al. 2020; Suberg et al. 2019). The Western English Channel exhibits thermal stratification during spring and summer, tidal dynamics as well induce many gyres in the Normand‐Breton Gulf (Hoch and Ménesguen 1997). The Central English Channel is influenced by river run‐off from both English and French coasts, including a permanent halocline stratification in the Bay of Seine created by inputs of the Seine and surrounding estuaries (Hoch and Ménesguen 1997). The eastern basin (French side Eastern English Channel) is characterised by the presence of bays and estuaries, where high proportions of terrestrial materials and brackish waters are discharged directly into the sea (Dauvin 2012) defining a Region Of Freshwater Influence (ROFI) separated from offshore waters by a frontal system called the ‘coastal flow’ (Brylinski et al. 1991). In addition, the English Channel (particularly by the Strait of Dover) is one of the busiest sea shipping routes, and vessel activities can also modulate the ecosystems' hydrological conditions (Glegg et al. 2015). All the above conditions lead to a wide range of ecological pelagic habitats along the English Channel.

Many observation and monitoring programs for coastal and offshore marine waters in the English Channel have been established for decades (Southward et al. 2005). However, most of the microbial taxonomical diversity surveys were focused on phytoplankton using microscopy (Widdicombe et al. 2010) and/or benchtop flow cytometry (Marie et al. 2010; Tarran and Bruun 2015). In recent years, by employing next generation sequencing (NGS) targeting rDNA genes, microbial community compositions of a few locations in the English Channel started to be explored at the molecular level (Lemonnier et al. 2020; Gilbert et al. 2009, 2012; Caracciolo et al. 2022; Rachik et al. 2018; Skouroliakou et al. 2024). Indeed, molecular approaches and NGS can provide valuable information on marine microbiota composition considerably and were recommended to be adapted as part of observation programs for further exploring/monitoring microbial diversities in the English Channel (López‐García and Moreira 2008; Goodwin et al. 2017). In the era of climate change, coastal habitats are strongly impacted by events such as ocean warming and acidification (Goberville et al. 2010; World Meteorological Organization, U.N 2023). Therefore, by using molecular approaches, this study aims to investigate the ecological and microbial succession, including environmental parameters and genetic‐level microbial diversity along coastal habitats in the French waters of the English Channel. This work aims to represent a baseline for exploring the feasibility of including bacterioplankton diversity into ecosystem models of coastal pelagic ecosystems and the possibility of using bacterioplankton diversity changes as a possible metric for calculating pelagic habitat and littoral water state indicators in addition to phytoplankton and zooplankton.

During the two ‘EcoPel’ oceanographic campaigns conducted during the two main productive periods in spring and summer 2018, biogeochemical data and sub‐surface seawater samples were collected in French waters of the English Channel from Brest to Dunkerque (in the North Sea). In this study, we extracted DNA from these samples and focused on analysing bacterial community compositions in various coastal habitats in two different seasons using the NGS‐metabarcoding approach. Furthermore, with the assistance of statistical tools, possible correlations between environmental parameters and the structure of marine microbiota were explored to provide a first understanding of the spatial and seasonal variability of bacterioplankton diversity.

## 2 Materials and Methods

### 2.1 Sampling Area and Strategy

**FIGURE 1:**
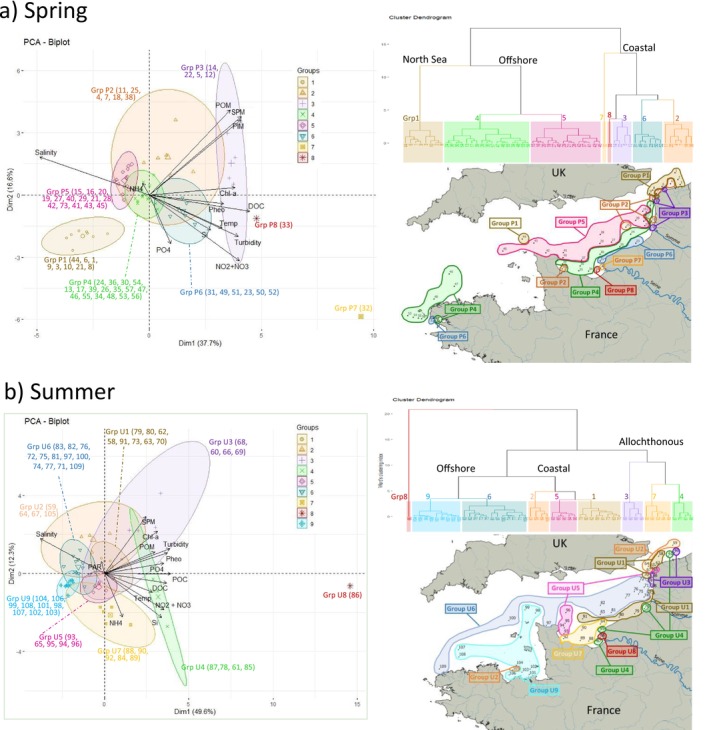
PCA environmental variables and clusters. Variability of environmental parameters during the ECOPEL 2018 oceanographic campaigns in (a) spring and (b) summer in surface waters of the French‐side English Channel. On the left side, Principal Component Analysis (PCA) applied to environmental variables recorded for each site of the oceanographic campaign. The PCA biplot represents the projection of the environmental variables (arrows) and the sampling sites (coloured symbols). Chl‐*a*: Chlorophyll‐*a*; NO_2_ + NO_3_: Nitrite + nitrate; NH_4_: Ammonium; Si: Silicate; PO_4_: Phosphate; DOC: Dissolved organic carbon; POM: Particulate organic matter; SPM: Suspended matter; PIM: Particulate inorganic matter; POC: Particulate organic carbon; Temp: Temperature, Pheo: Phaeopigments; PAR: Photosynthetically active radiation. The prediction ellipses displayed grouping of sample sites at 95% confidence. On the right side, the nine clusters of environmental parameters revealed by the Hierarchical Cluster Analysis (HCA) using Ward's method, and their spatial location in the English Channel in spring (a) and summer (b). The summer Groups U8 was also included into the ‘allochthonous’ category due to its location in the discharge area of the Seine River.

Sub‐surface seawater samples were collected during two exploratory oceanographic campaigns ‘EcoPel 2018’ (Artigas 2018) in French waters of the eastern English Channel onboard the ‘Antéa’ R.V. (IRD—French Oceanography Fleet): starting from Boulogne‐Sur‐Mer, covering waters from offshore Dunkerque (North Sea) to Brest (Iroise Sea) and making coast‐to‐offshore transects (Figure S1). The campaigns took place in spring (April 18–May 2, 2018) and summer (July 16–July 31, 2018). A total of 108 subsurface (2 m) seawater samples were collected using Niskin bottles, with 56 samples collected in spring (April 18–30, 2018) and 52 samples in summer (July 18–31, 2018; Figure 1). Seawater samples were immediately pre‐screened through a 150 μm mesh to remove large organisms (e.g., metazoans) and big colonies/particles, and then were filtered into 0.22 μm Sterivex cartridges (MilliporeSigma, Burlington, MA, USA) until the filter clogged. Filtered seawater volumes varied by samples, ranging from 142 mL (site 33) to 6500 mL (site 48). Sterivex cartridges were immediately wrapped with parafilm and stored at −80°C until DNA extraction.

Maps were generated using the QGIS v3.10.2 software (Neteler et al. 2012) with RGF93/L‐93 coordinate reference system. The ‘shapefiles’ used to create the reference map were taken from: www.gadm.org, www.naturalearthdata.com, www.old.atlas‐belgique.be and www.services.sandre.eaufrance.fr. Diversity estimators and taxonomic abundances (OTUs) were displayed using Inverse Distance Weighting (IDW) interpolation with a continuous gradient.

### 2.2 Characterisation of Environmental Variations

Physico‐chemical and biogeochemical data that were collected at 2 m depth for each sampling site are: temperature, salinity, turbidity, nitrate, nitrite, ammonium, silica, phosphate, as well as chlorophyll *a*, phaeopigments, dissolved organic carbon concentrations, particulate organic and inorganic matter, suspended matter and particulate organic carbon.

A CTD probe (SBE19, Sea‐Bird Scientific, USA) was deployed for retrieving Sea Surface Temperature (SST, °C) and salinity (S, PSU). Concentrations of nitrite (NO₂^−^, μmol L^−1^), nitrate (NO₃^−^, μmol L^−1^), phosphate (PO₄^3−^, μmol L^−1^) and silicate (SiOH₄, μmol L^−1^) were analysed using the Futura II Autoanalyser (AMS Alliance, Italy). Suspended Particulate Matter (SPM, mg L^−1^) was determined by the weight difference of pre‐combusted GF/F filters (0.7 μm) before and after filtration according to an established protocol of the SOMLIT network (https://www.somlit.fr). Particulate Organic Carbon and Nitrogen (POC and PON, μg L^−1^) were estimated using a Fisons CHN Analyser (Fison, Glasgow, UK). Chlorophyll‐*a* (Chl‐*a*, μg L^−1^) and phaeopigments (μg L^−1^) samples were extracted for 24 h in 90% acetone following the fluorimetric protocol (Aminot et al. 2004) using a 10‐AU fluorometer (Turner Designs, CA, USA).

### 2.3 DNA Extraction, Amplicon Library Construction and Illumina Sequencing

Total nucleic acids were extracted from Sterivex filters using the Qiagen AllPrep DNA/RNA Mini kit (Qiagen, Hilden, Germany) following the manufacturer's protocol, and DNA concentrations were measured with the Qubit fluorometer (Thermo Fisher Scientific, Waltham, MA, USA). Universal primers S‐D‐Bact‐0341‐b‐S‐17 (5′‐CCTACGGGNGGCWGCAG‐3′) and S‐D‐Bact‐0785‐a‐A‐21 (5′‐GACTACHVGGGTATCTAATCC‐3′) (Klindworth et al. 2013) were used to amplify the hypervariable V3‐V4 region of the prokaryote 16S rRNA gene. Amplicon library construction and Illumina MiSeq paired‐end (Reagent Kit v2, 2 × 250 bp) sequencing were performed by GenoScreen (Lille, France). Sequencing data have been submitted to the NCBI sequence read archive database (SRA accession: PRJNA1242094).

### 2.4 Sequences Processing

A total of 1,889,181 reads were obtained from Illumina sequencing and then were processed with the MOTHUR v1.44.1 program (Schloss et al. 2009) following the standard operating procedure (Kozich et al. 2013). Sequences were extracted and separated according to their index tag, de‐replicated to unique sequences and aligned against the SILVA database v138 (http://www.arb‐silva.de) (Quast et al. 2013). Suspected chimeras were removed by using the UCHIME program (Edgar 2010). After quality filtering, an average of 17,332 (± 3214) reads per sample were clustered into operational taxonomical units (OTUs) at a 97% similarity threshold (Behnke et al. 2011). Singletons, referring to OTUs that have a single representative sequence in the whole data set, were removed as these are most likely erroneous sequencing products (Behnke et al. 2011; Kunin et al. 2010). After normalization of the entire dataset, all remaining 1,058,920 reads were clustered into 10,896 OTUs, with an average size of 449 bp and searched against the SILVA database (Quast et al. 2013) by using BLASTN (Altschul et al. 1990). BLASTN results were carefully examined and manually curated for assigning putative taxonomic affiliations to each OTU. OTUs that were identified as Eukaryotes (e.g., chloroplasts) were removed from analyses to focus on bacterial diversity.

### 2.5 Data Analyses

#### 2.5.1 Environmental Characterisation of the Study Area

The association between stations and environmental variables was performed using Principal Component Analysis (PCA) with normalised environmental parameters using the ‘FactoMineR’ package (Lê et al. 2008) in R (v 4.0.1) (R Core Team 2021). Based on the obtained PCA biplots, the Euclidian distance matrices between sites were calculated. The dissimilarity matrices were then subject to hierarchical cluster analysis based on the Ward's minimum variance method (Ward Jr 1963) with ‘Ward.D2’ clustering method in the ‘hclust’ function of the ‘cluster’ R package (Murtagh and Legendre 2014). This method defines well‐delimited groups by minimising total within‐cluster variance (Ward Jr 1963).

#### 2.5.2 Microbial Diversity Assessment

Alpha diversity was calculated based on the Chao, Berger–Parker, Simpson and Shannon estimators using the Past 3.05 program (Hammer et al. 2001). The Chao (Chao1) richness index that estimates the theoretical number of expected OTUs was calculated based on the singletons and doubletons in each sample (Chao 1984). The Berger–Parker index gives the proportional abundance of the most abundant species in a given sample (Berger and Parker 1970). Simpson's index (1‐D) was based on the probability that two individuals belong to the same OTU: ranges from 0 (all taxa are equally present) to 1 (one taxon dominates in the community) (Simpson 1949). The Shannon equitability index (Lloyd and Ghelardi 1964; Pielou 1966; Shannon 1948) which reflects the evenness of the community, was calculated by dividing the Shannon diversity index (H′) by the maximum diversity (H'max).

Beta diversity analyses and visualisation were performed using the ‘R’ program (R Core Team 2021). The Bray–Curtis dissimilarity matrix (Bray and Curtis 1957), commonly used to calculate ecological distances based on OTUs (or species) abundances, was calculated on the square root transformed OTU read abundance using the ‘vegdist’ function from the ‘Vegan’ package of ‘R’ (Oksanen et al. 2020). Square root transformation aimed to reduce the dispersion between rare and very abundant OTUs. Microbial assemblages were grouped across sampling dates and locations. The dissimilarity matrices were then subject to hierarchical cluster analysis using the Ward method (Ward Jr 1963). From the dendrograms generated with hierarchical cluster analysis, the Dunn index (Dunn 1974) was calculated using the ‘NbClust’ function (Charrad et al. 2014) in order to obtain the optimal number of cluster groups. Samples from spring and summer were analysed separately, except when clustering the microbial community from the whole dataset. In order to determine the contribution (in %) that each OTU had on the formation of the different clusters, a similarity percentages (SIMPER) analysis was performed using the PRIMER v6.0 (Clarke and Gorley 2006).

### 2.6 Relationship Between Physicochemical/Biogeochemical Properties and the Most Abundant Bacterial Taxa

Influences of environmental parameters on the bacterioplankton community structure were investigated using both the canonical correspondence analysis (CCA) (ter Braak 1986) and Mantel correlation analysis (Mantel 1967). Using the ‘CCA’ function of the ‘Vegan’ package (Oksanen et al. 2020), relationships between environmental factors and bacterial taxa were examined. Significant correlations (*p* value < 0.05) between environmental parameters and the most abundant bacterial taxa were calculated using the ‘envfit’ function with 999 permutations (Han et al. 2019). After removing the non‐significant environmental factors and bacterial taxa, analysis of variance (ANOVA) was performed to test the significance of the new CCA models using the ‘Vegan’ package. Correlations between bacterial taxonomic composition and environmental parameters were evaluated by performing Mantel's analysis. First, Spearman's pairwise correlations on environmental variables were calculated, and then based on Euclidean distances, Mantel's correlations between taxonomic composition and environmental parameters were performed using the ‘Vegan’ package with 9999 permutations.

## 3 Results

### 3.1 Environmental Variables

Concentrations of nitrates and nitrite, particulate organic matter and suspended matter were higher in spring compared with summer, whereas, unsurprisingly, the sea surface temperature was higher during the summer campaign (Tables 1 and S1).

**TABLE 1:** List of environmental variables, minimal and maximal values, and their associated location sites during the two ECOPEL 2018 cruises (min–max, Site min–Site max).

Physicochemical parameters	Spring			Summer		
	Average (± std)	Values (min–max)	Sites (min–max)	Average (±std)	Values (min–max)	Sites (min–max)
Temperature (°C)	10.29 ± 0.94	9.01–12.35	1–51	18.43 ± 1.24	14.46–21.18	109–86
Salinity (psu)	34.10 ± 0.98	29.86–35.25	32–44	34.38 ± 0.81	30.51–35.20	86–107
Turbidity	1.10 ± 0.82	0.29–5.64	19–32	0.93 ± 0.71	0.26–3.96	105–86
Chl‐*a* (μg/L)	2.81 ± 2.53	0.23–13.01	27–33	1.57 ± 1.15	0.25–6.51	107–68
Phaeopigments (μg/L)	0.93 ± 0.64	0.11–2.55	45–23	0.44 ± 0.30	0.09–1.84	107–86
NO₂^−^ + NO₃^−^ (μmol/L)	4.20 ± 8.59	0.22–57.14	9–32	0.80 ± 1.04	0.15–7.09	67–86
NH_4_^+^(μmol/L)	0.56 ± 0.39	0.14–1.97	9–27	0.49 ± 0.39	0.02–2.15	76–61
Si (μmol/L)	0.87 ± 1.65	0.12–7.27	25–14	1.68 ± 1.62	0.25–9.34	73–86
PO_4_^3−^(μmol/L)	0.10 ± 0.09	0–0.48	21–32	0.02 ± 0.03	0–0.19	64, 82, 83, 90, 92–86
DOC (μmol/L)	83.30 ± 12.24	65.62–115.83	29–32	92.36 ± 12.10	74.07–130.83	105–86
POM (mg/L)	7.96 ± 3.42	1.43–18.08	44–5	1.01 ± 0.38	0.16–2.36	65–86
SPM (mg/L)	50.67 ± 10.07	4.78–93.64	1–5	1.90 ± 1.11	0.64–5.54	103–105
PIM (mg/L)	42.71 ± 16.09	3.21–75.57	1–5	—	—	—
POC (μg/L)	—	—	—	305.82 ± 177.27	128.49–1372.53	101–86

Abbreviations: Chl‐*a*, Chlorophyll‐*a*; NO_2_^−^, nitrite; NO_3_^+^, nitrate; NH_4_^+^, ammonium; Si, Silicate; PO_4_^3−^, phosphate; DOC, dissolved organic carbon; POM, particulate organic matter; SPM, suspended matter; PIM, particulate inorganic matter; POC, particulate organic carbon.

A principal component analysis (PCA) performed on environmental variables allowed distinguishing 8 and 9 different pelagic habitats during spring and summer, respectively (Figure 1). The two first dimensions of the PCA projection contributed to explain 54.3% and 61.9% of the total variability in spring and summer, respectively. In spring, suspended matter, particulate inorganic and organic matter, nitrate and nitrite, salinity, turbidity and dissolved organic carbon (ordered by increasing contribution) each contributed > 10% to the formation of the two dimensions of the PCA (Figure 1a). In summer, suspended matter, silicate, Chlorophyll‐*a*, salinity, nitrate and nitrite, turbidity, ammonium, phaeopigments, particulate and dissolved organic carbon (ordered by increasing contribution) each contributed > 10% to the formation of the two dimensions of the PCA (Figure 1b).

According to the result of hierarchical cluster analysis based on environmental variables, spring water masses could be separated into 8 pelagic habitats or water types that characterised three main clusters that corresponded to North Sea waters (group P1), ‘Coastal’ waters (groups P2, P6, P3, P8 and P7) and ‘Offshore’ waters (groups P4 and P5) (Figure 1a). North Sea waters (P1) were associated with low concentrations of suspended matter, particulate organic and inorganic matter and high salinity. The P2 corresponded mostly to coastal waters discontinuously located in the North Sea and Eastern English Channel that were characterised by high values of suspended matter, particulate organic and inorganic matter. Coastal waters located on the east side of the Eastern English Channel and North Sea belonged to P3 and were characterised by high concentrations of suspended matter, particulate organic and inorganic matter, dissolved organic carbon and chlorophyll‐*a*. P4 and P5 included offshore waters spreading out in the Western and Eastern English Channel, with relatively homogeneous biogeochemical variables except high salinity and ammonium concentrations. The P6 corresponded to coastal waters likely influenced by continental runoff, with high nutrient concentrations (silicate, phosphate, nitrate, nitrite and dissolved organic carbon), chlorophyll‐*a*, phaeopigments, turbidity and temperature. P7 and P8, each corresponding to only one station, were directly influenced by the Seine estuarine inputs of more extreme environmental values. Water types characteristic of P7, P8, P6, P4 and P5 highlighted the dilution of nutrients runoff and turbidity from the Seine River on a coastal‐offshore gradient (Figure 1a).

Summer stations could be separated into 9 pelagic habitats or water types according to environmental variables, and could be further categorised into: ‘Offshore’ waters (groups U6 and U9), ‘Coastal’ waters (groups U1, U2 and U5) and ‘Allochthonous’ waters (groups U3, U4, U7 and U8) that were directly influenced by river discharge and/or continual runoff (Figure 1b). The U1 corresponded to coastal Eastern English Channel and North Sea waters, and was not strongly associated with any particular environmental variable. However, U1 waters were characterised by high nutrient concentrations, Chlorophyll‐*a* and particles. The North Sea waters (U2) showed higher salinity and suspended matter charge. Noticeably, one station (105) from U2 was also located in the Côte d'Armour (Normand‐Breton Gulf) area. U5 waters were moderately influenced by environmental variables and river inputs. U3 and U4 waters spread along the coast of the Eastern English Channel and North Sea. These two groups were associated with high concentrations of suspended matter, chlorophyll‐*a*, particulate organic matter and turbidity (particularly for U3), and high concentrations of nutrients (phosphate, silicate, nitrate and nitrite), particulate and dissolved carbon, phaeopigments and higher temperature. Thus, these groups were assigned the name ‘allochthonous’ waters, suggesting direct strong influences from continental and river discharge of nutrients and matter. U7 and U8 waters were also included in the ‘allochthonous’ category due to their location in the discharge area of the Seine River. Finally, the ‘offshore’ category (U6 and U9) was characterised by high salinity and low concentrations of nutrients and organic matter. The U6 corresponded to Atlantic waters entering and circulating in the English Channel, while the U9 corresponded to the coastal‐offshore transect area surrounding the Channel Islands, the Normand‐Breton Gulf and the Côte d'Armour.

### 3.2 Overall Microbial Diversity

**FIGURE 2:**
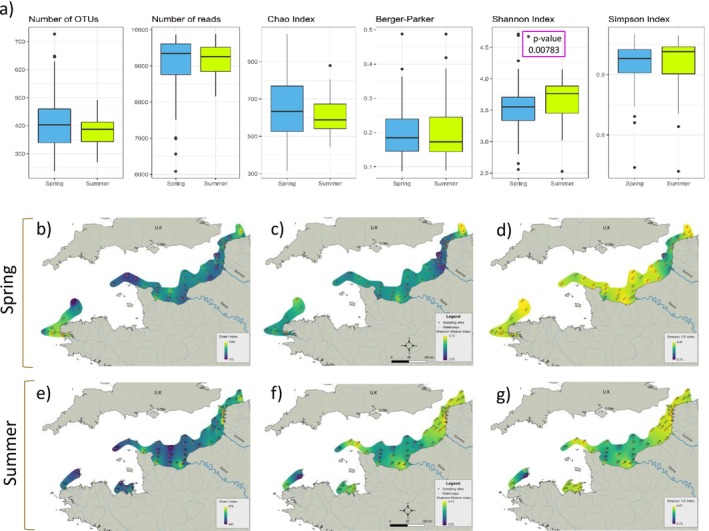
Alpha diversity indices. Bacterial alpha diversity in surface waters of the French‐side English Channel during the ECOPEL 2018 cruises. On the top part, (a) box plot representing variability of diversity indices in spring (in blue) and summer (in green). Lower part, spatial interpolations of diversity indices at each site for Chao in (b) spring and (e) summer, Shannon–Weaver's in (c) spring and (f) summer and Simpson's (1‐D) for (d) spring and (g) summer. The index values are indicated in colour gradient (with dark purple for low values and yellow for high values). For citations of indices, see the ‘Materials and Methods’ section, ‘Data analyses – Microbial diversity assessment’.

A total of 10,896 OTUs were identified during the two campaigns with 7229 OTUs in spring and 6243 OTUs in summer. Between spring and summer, 2576 OTUs (23.6%) were shared. On average, 396 (±82) OTUs were detected from each of the 108 samples collected in spring and summer (Figure 2a). Alpha diversity indices, including OTU numbers, Chao, Berger‐Parker and Simpson, suggested no significant differences (Mann–Whitney *p* value > 0.05) between the two sampling periods (Figure 2a). However, the richness and equitability Shannon index was significantly different during the two sampling periods (Mann–Whitney *p* value < 0.01) with high richness and equitability in summer compared to spring (Figure 2a). The Shannon (H′) and Simpson (1‐D) indices displayed similar spatial patterns on the same sampling period (Figure 2a). In spring, low richness and equitability were found in the area between Dieppe and Calais in the Eastern English Channel, with the lowest value recorded at station 16 offshore between the Bay of Somme and the Côte d'Opale (Figure 2b–d). On the opposite, higher richness and equitability were generally found in offshore waters of the Western English Channel, in the southern North Sea and in the bay of Seine, with hot spots at stations 1, 2, 34 and 46 (Figure 2b–d). In summer, patterns of richness and equitability appeared to be reversed with low values in offshore waters of the Western English Channel and the bay of Seine (except near the Seine River estuary) while higher values in the area ranging from Dieppe to the South part of the North Sea (Figure 2e–g). In addition, stations in the Normand‐Breton Gulf and Côte dArmor area displayed high Shannon and Simpson's values in summer (due to weather conditions during the spring campaign, this area was not sampled).

**FIGURE 3:**
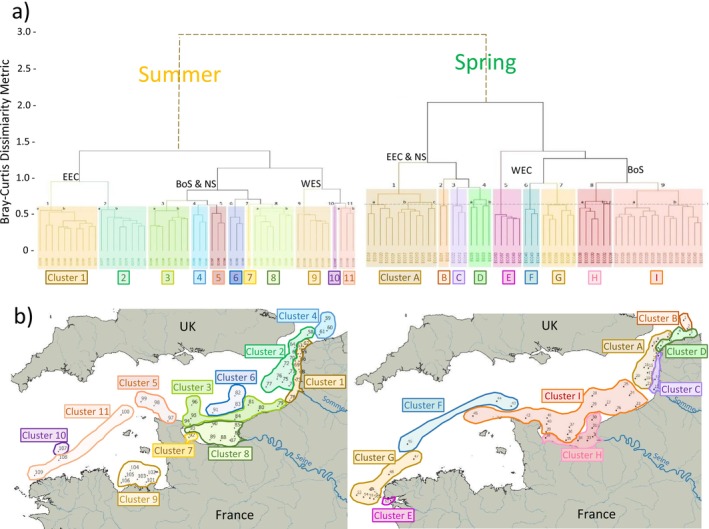
Structure of bacterial community. Structure of bacterial community in surface waters of the French‐side English Channel during the ECOPEL 2018 cruises. (a) Hierarchical Cluster Analysis (HCA) Cluster based on Ward's clustering method and Bray–Curtis dissimilarities dendrogram was calculated on the square root transformed number of reads for all (spring and summer) OTUs unveiled during the oceanographic campaigns. (b) Map of grouping sampling site according to HCA nine (spring, A–I) and 11 (summer, 1 to 11) clusters, with different colours representing different bacterial assemblages. Abbreviations: WEC: Western English Channel; BoS: Central English Channel‐Bay of Seine; EEC: Eastern English Channel; NS: North Sea.

The result of hierarchical cluster analysis performed on the entire dataset portrayed a clear seasonality in the bacterial community structure of the English Channel, with grouping of stations in the dendrogram according to the sampling season (Figure 3a). For the structure of the bacterial community, samples from the spring campaign were organised into 9 clusters and samples from the summer campaign were organised into 11 clusters (Figure 3b). These clusters clearly delimit different water masses where bacterial diversities were shaped by biogeochemical variables, currents and river discharges (Figures 1 and 3). In both seasons, the structure of the bacterial community could be characterised based on four geographic areas: Eastern English Channel, Western English Channel, Bay of Seine and south of the North Sea (Figure 3). Although grouped together as an individual cluster, stations in the North Sea cluster showed community structures closer to stations in the Eastern English Channel cluster in spring, but to stations in the Bay of Seine in summer. Interestingly, the Somme River estuary seems to delineate the Eastern English Channel into two parts: the south part under influences of the Seine River and the north part displayed different layers of bacterial communities structured around a gradient from coastal to offshore waters.

### 3.3 Taxonomic Diversity

During the two sampling periods, a total of 30 main bacterial taxonomic groups were detected. The major two groups, together representing 73.5% of all reads, were: Proteobacteria (mainly alpha‐ and gamma‐proteobacteria), which represented 45.6% of all reads, and Bacteroidetes (mainly Flavobacteriales, and a minority of Bacteroidales, Chitinophagales and Cytophages), representing 27.9% of all reads. Other groups that showed more than 0.1% of all reads included Actinobacteria (9.4% of the reads), Cyanobacteria (11.2%), Planctomycetes (0.9%) and Verrucomicrobia (4.7%).

### 3.4 Community Structure in Spring

**FIGURE 4:**
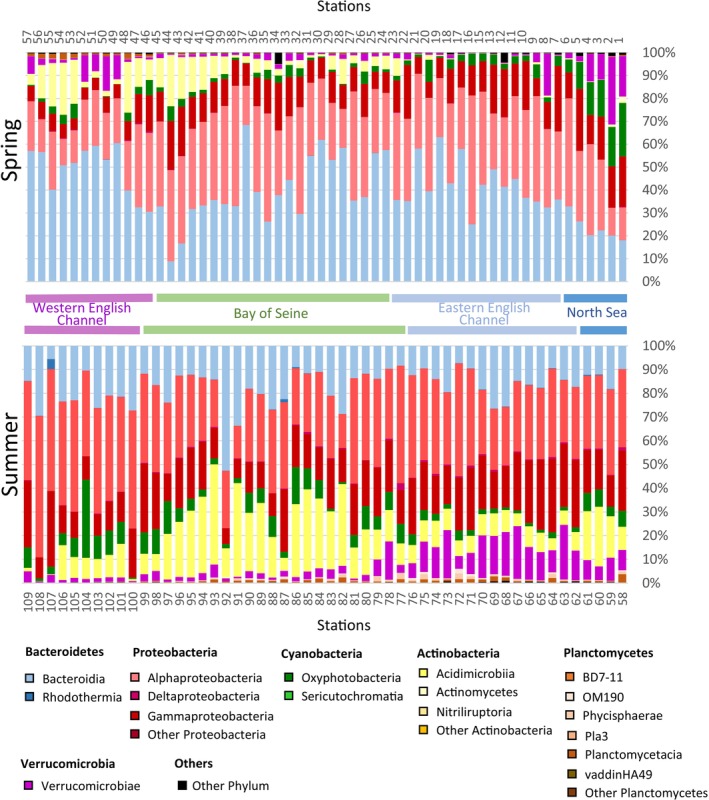
Bacterial community composition. Bacterial taxonomic (order level) composition of each sample taken during the ECOPEL 2018 cruises in spring (top part) and summer (lower part) in surface waters of the French‐side English Channel. Corresponding main areas are shown between the two graphs. ‘Other Phylum’ correspond to phyla that represent less than 0.5% of the reads at one season.

In spring, the distribution of major bacterial taxonomic groups (> 0.5% of total reads) displayed clear spatial patterns (Figures 4 and S2). The vast majority of the phyla found in the Bay of Seine belonged to Proteobacteria and Bacteroidetes. Higher proportions of Cyanobacteria and Verrucomicrobia were observed off Dunkerque and in the southern part of the North Sea. The Western English Channel had higher proportions of Actinobacteria compared with the Eastern English Channel.

*Proteobacteria* (41.1% of the spring community) was the most abundant phylum, composed of Alpha‐proteobacteria (71.1% of all Proteobacteria reads), Gamma‐proteobacteria (28.7%) and Delta‐proteobacteria (0.2%). In the English Channel, the proportion of Proteobacteria was relatively constant and corresponded to around 50% of all bacterial reads. However, lower proportions of Proteobacteria were found in the southern part of the North Sea and at the tip of the Bretagne peninsula (Iroise Sea and Finistery) (Figures 4 and S2).

*Bacteroidetes* (38.2% of the spring community) was the second most abundant phylum, with the Flavobacteriales order composed of the vast majority (97.5% overall), while Chitinophagales, Cytophagales and Bacteroidales remained at lower abundances (all above orders belong to the class of Bacteroidia, Figure 4 and Table S1). In the southern part of the North Sea, Bacteroidetes displayed a lower abundance and then abundances increased with a gradient southward into the Eastern English Channel. Although at low abundances overall, Chitinophagales and Cytophagales took up a higher proportion of Bacteroidetes abundance in the southern part of the North Sea, and their proportions were decreased with a gradient southward (Figure 4 and Table S1). Off the mouth of the Seine River (station 34), a higher proportion of Chitinophagales and Bacteroidales was observed (together representing around 20% of total Bacteroidetes reads) (Figures 4 and Table S1). In the region of the bay of Seine and in the Western English Channel, Bacteroidetes showed lower abundances in general, with the lowest number of reads observed offshore the Cotentin peninsula (stations 43 and 44) and read numbers increasing westwards (stations 45–49) (Figure 4 and Table S1). In this area (station 44), a higher proportion of Cytophagales was observed, representing 16% of all Bacteroidetes reads (Table S1). Finally, at the extremity of the Bretagne peninsula where Atlantic waters enter the Iroise Sea near Brest (stations 49–57), higher abundances of Bacteroidetes (mainly composed of Flavobacteriales) were observed (Figure 4 and Table S1).

*Cyanobacteria* (11.4% of the spring community) represented higher proportions in bacterial reads in the southern part of the North Sea as compared with other locations (Figure 4 and Table S1). Within the phylum of Cyanobacteria, two classes Oxyphotobacteria (majority) and Sericytochromatia (minority) were identified.

*Actinobacteria* (5.2% of the spring community) accounted for higher proportions of bacterial reads near the Bretagne peninsula (stations 39–48) and the Cotentin peninsula (stations 53–56) as compared with other locations (Figure 4 and Table S1). Within the phylum of Actinobacteria, Microtrichales and Actionomarinales (in the class of Acidimicrobiia) were the two most abundant orders generally, except for station 32 at the mouth of the Seine (with higher Frankiales in the class of Actinomycetes), also in the south part of the North Sea (stations 1–7) and near Boulogne‐sur‐mer (stations 12–13) (with diverse taxonomic orders) (Figure 4 and Table S1).

*Verrucomicrobia* (3.2% of the spring community) was more represented in the waters of the southern part of the North Sea and near Brest as compared with other locations (Figure 4 and Table S1). Within the phylum of Verrucomicrobia, Opitutales and Verrucomicrobiales (in the class of Verrucomicrobiae) were the two most abundant orders generally, except for stations in the Iroise Sea and the bay of Brest where Verrucomicrobiales were observed in very high proportions (stations 49–57) (Figure 4 and Table S1).

### 3.5 Community Structure in Summer

In summer, most OTUs were distributed among 6 different phyla: Actinobacteria, Bacteroidetes, Cyanobacteria, Planctomycetes, Proteobacteria and Verrucomicrobia. Generally, Proteobacteria and Bacteroidetes were (as in spring) the two most abundant phyla, together representing two thirds of all summer reads. However, as compared with the spring community, Actinobacteria, Planctomycetes, Proteobacteria and Verrucomicrobia proportions in the summer community increased while Bacteroidetes proportion decreased (only reaching 50% of total reads at station 93). High proportions of Planctomycetes and Verrucomicrobia were observed in the Eastern basin of the English Channel, whereas a higher proportion of Actinomycetota was observed in the Bay of Seine. However, the lowest read was recorded at station 92 in the western Bay of Seine (Bay of Veys) (Figures 4 and S2).

*Proteobacteria* (45.6% of the summer community) was the most abundant phylum. Particularly, Proteobacteria accounted for higher proportions of the summer community in the Western English Channel as compared with the spring community (Figure 4 and Table S1). Within the phylum of Proteobacteria, higher proportions of Gamma‐proteobacteria and Delta‐proteobacteria were observed in the Eastern English Channel and Bay of Seine (except station 92 in the Bay of Veys) (Figure 4 and Table S1).

*Bacteroidetes* (16.6% of the summer community) was the second most abundant phylum, although its proportion in the community significantly decreased as compared with spring (Figure 4). Overall, more than 90% of the Bacteroidetes belonged to the Flavobacteriales order except for stations 68, 82 and 107. Besides Flavobacteriales, Sphingobacteriales was mostly found in the Eastern English Channel while Chitinophagales was mostly observed in the Bay of Seine (all above orders belong to the class of Bacteroidia, Figure 4 and Table S1). Notably, at station 107 offshore of the Côte des Abers, a community composition with the lowest proportion of Flavobacteriales and the highest portion of Balneolales (in the class of Rhodothermia) was observed (Figure 4 and Table S1).

*Actinobacteria* (14% of the summer community) occupied higher proportions in summer communities in the North Sea, the Eastern English Channel and the Bay of Seine as compared with spring communities (Figure 4 and Table S1). For most stations, more than 90% of Actinobacteria belonged to the order Actinomarinales (in the class of Acidimicrobiia) except for station 108, where the community was composed of 70% Actinomarinales, 20% Nitriliruptorales (in the class of Nitriliruptoria), and 10% Microtrichales (in the class of Acidimicrobiia) (Figure 4 and Table S1).

*Cyanobacteria* (11% of the summer community) showed higher proportions in summer communities in the Western English Channel and lower proportions in the North Sea, opposite of the spring distribution (Figure 4 and Table S1). Only the class of Oxyphotobacteria was identified within Cyanobacteria in all summer samples.

*Verrucomicrobia* (6.3% of the summer community) was more represented in summer communities of the Eastern English Channel (compared to spring, and also compared to the Western English Channel) (Figure 4 and Table S1). Generally, within the phylum of Verrucomicrobia, Opitutales and Verrucomicrobiales were the two most abundant orders, and stations 82 and 92 had the highest proportions of Verrucomicrobiales (> 95%). Additionally, in the Bay of Seine (except station 92) and station 109 in Côte des Abers, high proportions of Pedosphaerales were observed (all above orders belong to the class of Verrucomicrobiae, Figure 4 and Table S1).

*Planctomycetes* (1.4% of the summer community) was more represented in summer communities in the Eastern English Channel and the Bay of Seine (compared to spring, and also compared to the Western English Channel) (Figure 4 and Table S1). Most of Planctomycetes belonged to classes of Planctomycetacia, Phycisphaerae and the Planctomycetes OM190 clade. Notably, for station 107 at the mouth of the Bay of Veys, the Planctomycetes community was fully composed of the Planctomycetes OM190 clade (Figure 4 and Table S1).

### 3.6 Beta Diversity

#### 3.6.1 Dissimilarity of Bacterial Assemblages

Based on the hierarchical cluster analysis applied to the whole data sets, the result clustered all samples into two distinct groups. Interestingly, one group contains samples exclusively from spring and the other group contains samples exclusively from summer (Figure 3a). This result suggested significant influences of seasonal factors on bacterial assemblages and community structures. As shown in the dendrogram, nine main clusters (cluster A–I) were categorised under the spring group while 11 clusters (cluster 1–11) were categorised under the summer group (Figure 3). In contrast to stations in certain biogeochemical clusters that were scattered in places (e.g., Groups P2 and U4, Figure 1), stations in the same or closer community cluster (based on the Bray–Curtis dissimilarity metric) all appeared to be in the vicinity (Figure 3). For example, cluster H and I in the spring group were all from the Bay of Seine area, and cluster 1 and 2 in the summer group were all from the Eastern English Channel (Figure 3). This result implied influences of geographic/environmental factors on bacterial assemblages and community structures, even though their correspondence to water types or pelagic habitats is not always easy to make.

#### 3.6.2 Contribution of OTUs in Each Cluster

To determine the contribution of each OTU to cluster formation, SIMPER analyses were performed with inputs of the clustering results (Figures 3 and S3). In spring, Clusters A, D (coastal North Sea and offshore Eastern English Channel) and H (coastal Bay of Seine) were mostly influenced by OTU1 (*Sulfitobacter*) (9.92%, 8.32% and 7.38% respectively). Cluster B (offshore North Sea) was influenced by OTU17 (*Persicirhabdus*, 4.7%). Cluster C (coastal waters of Côte d'Opale) was influenced by OTU5 (*Polaribacter*, 9.49%) and OTU27 (*Sulfitobacter*, 7.84%). In the Bay of Brest, Cluster E was influenced by OTU7 (Cryomorphaceae, 4.94%), OTU9 (Flavobacteriaceae_NS5, 4.78%), OTU6 (*Planktomarina*, 4.57%) and OTU5 (*Polaribacter*, 4.05%). Cluster F (offshore mid‐Western English Channel waters) was mostly influenced by OTU1 (*Candidatus_Actinomarina*, 6.08%) and OTU3 (SAR11‐Ia, 5.78%). Cluster G, stretching from the Iroise Sea to offshore the Côte des Abers, was mainly influenced by OTU1 (*Candidatus_Actinomarina*, 5.85%) and two different Flavobacteriaceae_NS5 (OTU9, 4.76% and OTU8, 4.29%). Finally, the biggest assemblage, Cluster I (including complete coastal‐offshore gradients off the Côte d'Albâtre, offshore waters of the Bay of Seine, Cotentin and Channel Islands) was mostly influenced by OTU2 (*Suflitobacter*, 4.8%), OTU1 (*Candidatus Actinomarina*, 3.89%), OTU6 (*Planktomarina*, 3.79%) and two different Flavobacteriaceae_NS5 (OTU8, 4.15% and OTU9, 3.87%).

On the other hand, clusters in summer were mostly influenced by one or a combination of the following three OTUs at different levels: OTU1 (*Candidatus_Actinomarina*), OTU3 (SAR11‐Ia) and OTU4 (SAR86) (Figures 3 and S3). The exception was Cluster 11, which followed the route of Atlantic waters entering the Western English Channel and was greatly influenced by OTU2 (*Sulfitobacter*, 11.37%). Cluster 7 (coastal waters off the Bay of Veys in the Bay of Seine) and Cluster 10 (offshore the Côte des Abers in the Western English Channel) were only represented by one station (respectively 92 and 107); therefore, the SIMPER analysis could not be performed.

### 3.7 Correlations Between Physicochemical Properties and Bacterial Diversity

**FIGURE 5:**
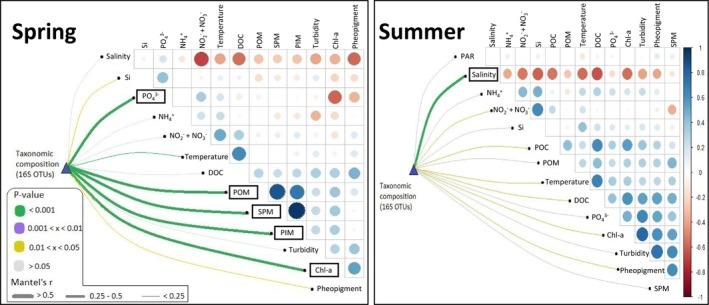
Environmental drivers of bacterial community composition. Pairwise comparison of environmental variables and bacterial taxonomic in surface waters of the French‐side English Channel composition in spring (left side) and summer (right side) during the ECOPEL 2018 cruises. The heatmap shows pairwise correlation between environmental variable, based on Spearman's correlation, with red and blue representing negative and positive correlations (scale indicated on the right side of the figure), while the significant correlation levels among dependent variables are indicated by circle diameter (larger circle size representing higher correlation values between two factors). Mantel tests quantified the correlation between bacterial taxonomic community composition and each environmental variable. Edge width represents the Mantel's r statistic for the corresponding distance correlation, and edge colour denotes the statistical significance (*p* value) based on 9999 permutations.

Correlations between taxonomic composition and environmental parameters were evaluated using Mantel's test, and relationships between environmental factors and bacterial taxa were examined with Canonical Correspondence Analysis (CCA) (Figures 5 and S4).

In spring, based on distance correlations and the statistical significance, environmental properties that were strongly correlated with the taxonomic composition included: chl‐*a*, PO_4_, POM, SM and PIM (*p* value < 0.001; correlation coefficient *r* ranged from 0.25 to 0.5) (Figure 5). Additionally, temperature, salinity, turbidity, PO_4_, phaeopigments and chl‐*a* were significant explanatory factors that might have driven the observed spatial distribution of taxa represented in the CCA (Figures 5 and S4). Overall, influences of environmental factors on taxa distribution in the English Channel were reflected in the CCA results (Figure S4). In spring, Actinomarinales, *Synechococcus* and Deltaproteobacteria were influenced by high values of salinity. Pedosphaerales were influenced by high concentrations of PO_4_ as well as low values of temperature, phaeopigments and chl‐*a*. Corynebacteriales, Propionibacteriales, Pseudonocardinales, Opitutales and Euzebyales were influenced by high turbidity, chl‐*a*, phaeopigments, as well as low values of temperature and salinity. Verrucomicrobiales and Microtrichales were influenced by temperature.

In summer, on the contrary, salinity was the only parameter strongly correlated with taxonomic composition according to results of Mantel's test (Figures 5 and S4). For the distribution of taxa, results of CCA suggested that salinity, turbidity, DOC, SPM, Si, NO_2_ and NO_3_, phaeopigment and chl‐*a* were significant explanatory factors (Figures 5 and S4). Opitutales, Verrucomicrobiales, Sphingobacteriales and Actinobacteria PeM15 were influenced by high turbidity, SPM, DOC, phaeopigments, chl‐*a*, as well as low values of Si and NO_2_ and NO_3_ (Figure S4). In contrast, Pedosphaerales was strongly correlated with NO_2_ and NO_3_, while Actinomarinales and Chitinophagales were influenced by high values of Si as well as NO_2_ and NO_3_. Finally, Balneolales was influenced by Salinity.

## 4 Discussion

### 4.1 Environmental Influences

Physicochemical and biogeochemical variables in marine and coastal systems are influenced by environmental changes such as seasons (e.g., temperature, light intensity), wind, rainfall, river flow, tides, currents and human activities (Cooley et al. 2022). In this study, river runoff seems to be one of the major factors that contribute to environmental variability. For both spring and summer, stations near the mouths of the Seine River and the Somme River estuaries always showed high values of NO_2_, NO_3_, PO_4_, Si, DOC and turbidity and lower values of salinity. Particularly, stations 32 (spring) and 86 (summer) located in coastal waters off the Seine estuary registered the highest concentrations in both inorganic nutrients and organic compounds, and the lowest values of salinity. Offshore these estuaries, concentrations of NO_2_, NO_3_ and PO_4_ were higher in spring than in summer, likely influenced by higher river discharge and continental runoff in early and mid‐spring. Nitrogen and phosphorus are key nutrients for algae and phytoplankton growth. In the English Channel, recurrent phytoplankton blooms such as diatom blooms in the Bay of Seine (Videau et al. 1998; Brunet et al. 1996) and *Phaeocystis* blooms in Côte d'Opale and the Bay of Somme (Spilmont et al. 2009; Lefebvre and Dezécache 2020) are well documented. In the spring dataset, high values of Chl‐*a* and phaeopigments were indeed observed in the Bay of Seine, the Bay of Somme and the Côte d'Opale (Table S1), suggesting phytoplankton blooms. As an important part of the marine nutrients cycle, phytoplankton blooms lead to a boost in biogeochemical activities and subsequently promote the activity of heterotrophic bacteria in the microbial loop (Buchan et al. 2014). The microbial community reacts accordingly to such seasonal/transient events with dynamics and re‐balance in gene expressions and community structures (Azam et al. 1983; Buchan et al. 2014). A recent study has started to explore associations between blooming phytoplankton and microbial communities in coastal waters of the Eastern English Channel (Skouroliakou et al. 2024). According to metabarcoding results, in spring the most abundant taxa at the mouth of the Seine River were *Sulfitobacter*, *Polaribacter* and *Planktomarina*, while the most abundant taxa at the mouth of the Somme River and the Côte d'Opale were *Polaribacter*, *Sulfitobacter* and *Glaciecola* (Table S1 and Figure S3). All the above taxa were previously reported to be associated with phytoplankton blooms (Beiralas et al. 2023; Hahnke et al. 2015; Wemheuer et al. 2015; von Scheibner et al. 2017), and *Glaciecola* was also reported to be associated with elevated levels of terrestrial runoff and DOM (Rodríguez et al. 2018). However, further studies are needed to understand what roles these taxa play in the microbial loop, how microbial communities react/interact with phytoplankton, convert phytoplankton biomass and their derived organic matter into bacterial biomass, and whether the bacterial biomass ends up entering the marine food web, returning to the atmosphere as CO_2_, or sinking down through the biological pump into long‐term storage.

In addition, according to the results of hierarchical cluster analysis for environmental variables (Figure 1), except for small spotty clusters or some coastal‐offshore transects off Dunkerque (spring) or Normand‐Breton Gulf (summer), most clusters seem to align to the coast. For example, as shown in Figure 1a, the spring water types P6, P4 and P5 (from inshore to offshore) are parallel to the French coast in the Eastern English Channel. The English Channel is known for strong tidal currents and a wide tidal range. Driven by tidal waves from the Atlantic Ocean and south‐west winds, the residual circulation drifts north–eastward. Along the French coast, estuarine influences combined with tide currents generate a nearshore water mass, the ‘coastal flow’ (Brylinski et al. 1991), a Region of freshwater influence (ROFI) separated from the open sea by a frontal area. It is possible that the appearance of coast‐aligning environmental clusters (Figure 1) was the consequence of ‘coastal flow’ influences. In an alternative way, the result of our cluster analysis for biogeochemical variables seems to agree with the observation of this ‘coast flow’. Previous studies have shown that tidal currents can also have influences on sediments (Reynaud et al. 2003) and storm surges (Idier et al. 2012) that may also, at least partially, contribute to the physicochemical and biogeochemical clustering.

### 4.2 Portraying Bacterial Clusters—Spring

Located between Dieppe and Calais, spring bacterial clusters A (offshore) and C (coastal) were dominated by phyla Bacteroidetes and Proteobacteria (mostly Alpha‐ and Gammaproteobacteria), and this might contribute to the observation of lower index values in ‘species’ evenness. Bacteroidetes and Proteobacteria were reportedly abundant during phytoplankton blooms (Buchan et al. 2014; Fandino et al. 2001; Pinhassi et al. 2004; Rink et al. 2007). Particularly, Lamy et al. (Lamy et al. 2009) have observed that Alpha‐ and Gammaproteobacteria, as well as Bacteroidetes, were the most important bacterial groups during the spring blooms of *Phaeocystis globosa* in the Eastern English Channel and contributed to leucine incorporation, which correlated with exo‐proteolytic and exo‐glucosidic activities. Skouroliakou et al. (Skouroliakou et al. 2024) have reported that dominant orders during the *P. globosa* bloom were affiliated with Flavobacteriales, Rhodobacteriales, Cellvibrionales and Opitutales. Above findings suggested that the bacterial diversity of both spring clusters A and C was influenced by phytoplankton blooms, although these two clusters were dominated by different strains/species of *Sulfitobacter* (Alphaproteobacteria) (Figure S3). The *Sulfitobacter* strain/species that influenced cluster A also influenced cluster D (coastal NS waters from Wissant to Dunkerque) and cluster H (Seine directly‐influenced coastal waters at BoS). Most *Sulfitobacter* species possess enzymes facilitating sulfite oxidation (Xu et al. 2024), and certain species reportedly could protect phytoplankton *Emiliania huxleyi* (which is a haptophyte, as is *Phaeocystis globosa* ) from pathogenic bacteria (Beiralas et al. 2023). On the other hand, previous studies had reported that some *Sulfitobacter* strains could, on the contrary, be responsible for algicidal effects on haptophytes by metabolizing algal DMSP to produce high amounts of methanethiol (Barak‐Gavish et al. 2018). We could infer that there might be certain mechanisms and associations between the haptophytes and *Sulfitobacter*.

Located at the southern part of the North Sea, spring bacterial cluster B recorded the highest values of diversity and evenness and was abundant in Verrucomicrobia and Bacteroidetes. According to the results of the SIMPER analysis, this cluster was mostly influenced by *Persicirhabdus* (Verrucomicrobia). *Persicirhabdus* was often found to be associated with shellfish microbiomes (Biessy et al. 2020) and was previously reported to be positively correlated with Tetrodotoxin (TTX) concentrations in TTX‐bearing *Crassostrea gigas* (Biessy et al. 2024).

Clusters I, F and G, widely distributed from offshore (and sometimes also coastal) Côte d'Albâtre and the Cotentin Peninsula, open waters of the Western English Channel offshore Normand‐Breton Gulf, to offshore the Finistery and the Iroise Sea (Figures 3 and S1), were associated with less charged SPM and turbidity as compared with waters in the Eastern English Channel. These clusters were influenced by *Candidatus Actinomarina*. *Ca*. *Actinomarina* contain rhodopsin, suggesting that these cells could rely on a photoheterotrophic lifestyle (Ghai et al. 2013). Additionally, cluster F was also influenced by SAR11, the most abundant planktonic microorganism in the oceans. SAR11 marine bacteria require exogenous reduced sulphur (e.g., dimethylsulfoniopropionate (DMSP), methionine) for growth (Tripp et al. 2008), and have the ability to both oxidise and produce a variety of volatile organic compounds (e.g., dimethyl sulfide (DMS), methanethiol) that can diffuse into the atmosphere (Giovannoni 2017). DMSP can be produced by marine phytoplankton such as *Phaeocystis* and *Emiliania huxleyi* (Yoch 2002). A previous study has found that *Emiliania huxleyi* bloom‐associated prokaryotic communities were dominated by SAR11 (Câmara Dos Reis et al. 2023).

At the roadstead of Brest, cluster E was influenced mainly by four OTUs: *Polaribacter*, Cryomorphaceae, Flavobacteriaceae_NS5 and *Planktomarina*. Among them, Cryomorphaceae and *Planktomarina* only influenced cluster E. Cryomorphaceae was previously found to be associated with phytoplankton (Pinhassi et al. 2004) while some Cryomorphaceae may exhibit photoheterotrophy due to the presence of proteorhodopsins (Gómez‐Consarnau et al. 2019). *Planktomarina* can also benefit from light and carbon monoxide oxidation as complementary energy sources when under severe starving conditions (Giebel et al. 2019). Regarding Flavobacteriaceae_NS5, this group influenced cluster E and cluster G, which are located next to Brest in the Iroise Sea and offshore the Finistery (Figure 3 and S1). Flavobacteriales was reported in highest abundances during the decay phase of phytoplankton blooms (Buchan et al. 2014; Pinhassi et al. 2004; Riemann et al. 2000), and was suggested to play a role involving the transformation of high molecular weight compounds into low molecular weight compounds (Teeling et al. 2012).

### 4.3 Portraying Bacterial Clusters – Summer

According to the result of the SIMPER analysis, most of the summer bacterial clusters (except Cluster 7 and 10) were influenced by *Candidatus Actinomarina*, *Sulfitobacter*, clade SAR11 and clade SAR86 (Figure 3 and S3). In contrast to the observations in spring, the summer bacterial Cluster 1 and 2 located between Dieppe and Calais displayed high values of ‘species’ richness and evenness. The result of the SIMPER analysis also showed that Cluster 1 and 2 were influenced by more OTUs as compared with other clusters. Onshore of the Côte d'Opale, Cluster 1 was influenced by SAR86, *Ca. Actinomarina*, SAR11, *Lentimonas* and *Roseibacillus*; offshore of the Côte d'Opale, Cluster 2 was only influenced by *Ca. Actinomarina*, SAR11 and SAR86. Besides *Ca. Actinomarina* and SAR11 that were mentioned above, some members of the SAR86 clade possess the ability to use light directly for adenosine triphosphate (ATP) production (Béjà et al. 2000), and can be associated with phytoplankton growth and algal blooms (Buchan et al. 2014; Seyedsayamdost et al. 2011). In addition, certain marine Verrucomicrobia including *Lentimonas* were previously reported with abilities to degrade algal polysaccharide such as fucoidan (Sichert et al. 2020; Zhang et al. 2024). Further investigation incorporating the diversity of phytoplankton/algae will help to paint a more complete picture for interactions between bacterial communities and phytoplankton/algae blooms, as well as on the biogeochemical processes (e.g., carbon cycle, (Freitas et al. 2012)) they might be involved.

The SIMPER analysis could not be performed for summer Cluster 7 and Cluster 10 due to each cluster only containing one station. Therefore, the most abundant OTUs in these two clusters were considered. Directly at the mouth of the Bay of Veys, station 92 (Cluster 7) was abundant in Cryomorphaceae, Flavobacteriaceae_NS4 and *Ca. Actinomarina*. According to analyses of genome sequences, certain photoheterotrophic members in Cryomorphaceae encode genes for the degradation of proteins and algal storage polysaccharides (Grieb et al. 2020). A previous report had a description of seasonal niches for the NS4 marine group before and/or after major phytoplankton bloom events in temperate marine systems (Alonso‐Sáez et al. 2015). Offshore the Côte des Abers where Atlantic waters enter the Western English Channel, station 107 (Cluster 10) was abundant in clade SAR86 and *Candidatus Puniceispirillum*. The SAR116 clade member *Ca. Puniceispirillum* was reportedly with phototrophic potential under nutrient‐limited conditions and could have a DMSP‐enhanced growth response when cultured with C1 compounds (Lee et al. 2019).

## 5 Conclusion

This study had surveyed the entire English Channel for environmental variables and bacterial communities during two distinct seasons. A total of 108 samples (56 samples from spring and 52 samples from summer) collected during the two ‘EcoPel’ oceanographic campaigns were analysed for bacterial community composition in addition to environmental variables. Results of PCA suggest SPM, POM, PIM, salinity and NO_2_^−^/NO_3_^+^ contributed most to the environmental characterization of water types in spring, while salinity, SPM, Si and Chl‐*a* contributed most to the environmental characterization of water types (pelagic habitats) in summer. The alpha diversity Shannon index suggested summer had higher richness and equitability compared to spring, while results of hierarchical cluster analysis indicated a clear seasonality in the bacterial community structure. Results of 16S rDNA sequencing revealed that Proteobacteria, Bacteroidetes, Cyanobacteria, Actinomycetota and Verrucomicrobia comprised the most represented taxa in both seasons. When compared with the spring community, the summer community showed a lower proportion of Bacteroidetes and a higher proportion of Proteobacteria, as well as Actinomycetota, Verrucomicrobia and, to a lesser extent, Planctomycetes. Based on distance correlations and statistical significance, the spring taxonomic composition was strongly correlated with Chl‐*a*, PO_4_, POM, SPM and PIM, while the summer taxonomic composition was only strongly correlated with salinity. Notably, both results of cluster analyses for biogeochemical variables and bacterial communities showed that most clusters seemed to be in parallel with the coast implying possible influences from the ‘coastal flow’. In addition, putative characteristics of influential taxa in certain bacterial clusters (results of the SIMPER analysis) suggested possible relations between bacterial communities and phytoplankton/algae dynamics (e.g., algae blooms). These seasonal differences in bacterial composition likely reflect their ecological roles. In spring, Bacteroidetes and Proteobacteria (Alpha‐ and Gammaproteobacteria) dominated coastal and offshore clusters, likely driven by phytoplankton blooms such as *Phaeocystis globosa* . These groups are key in recycling organic matter and are linked to exo‐enzymatic activities that degrade high molecular weight compounds. *Sulfitobacter*, in particular, may either support haptophytes by offering protection against pathogens or control their growth through an algicidal effect. In summer, Actinomycetota like *Candidatus Actinomarina*, along with SAR11, SAR86 and *Lentimonas*, were more abundant in oligotrophic waters, reflecting their roles in photoheterotrophy, nutrient cycling and degradation of algal polysaccharides.

For the future prospect of marine environment monitoring, incorporating molecular biology methods/analyses and hydro‐biogeochemical measurements for long‐term wide spatial surveys will help to paint a more complete picture of marine biodiversity, further understanding its influences on biogeochemical cycles and potentially conveying detrital organic matters into food webs, related potential biological and chemical interactions, water quality and ecosystem progress in this ever‐changing world. In addition, our results represent an Essential Ocean Variable that will be considered within the Global Ocean Observing System as ‘Microbe Biomass and Diversity’ for better integration of bacterial diversity into the calculation of indicators of the status of marine pelagic ecosystems and models as supports of environmental national and international policies.

## Author Contributions

S.M. and L.F.A. conceived of the presented idea, acquired funding, coordinated the cruise and performed sampling. S.M. and L.‐L.L. performed the sample processing. S.M., L.‐L.L. and N.D. carried out data analyses and results interpretation. S.M. and L.‐L.L. wrote the manuscript. L.F.A. and Z.H. provided critical feedback on the manuscript and results interpretation.

## Conflicts of Interest

The authors declare no conflicts of interest.

## Supporting information


**Figure S1:** Map of the study area during the ECOPEL 2018 cruises.
**Figure S2:** Taxonomic composition at each station in surface waters of the French‐side English Channel during the ECOPEL cruises. ‘Others’ represents all phyla that have their highest proportion lower than 1%.
**Figure S3:**. Heatmaps of the OTUs identified by SIMPER analysis as contributing the most (> 1%) to the clusters observed in (a) spring and (b) summer. The contributions are indicated with a colour gradient (see scale on the right side of the figure) with light yellow corresponding to low percentage of contribution and red to high contribution. (a) Bacterial contribution of each cluster assembly in surface waters of the French‐side English Channel during the ECOPEL 2018 spring cruise (SIMPER results). (b) Bacterial contribution of each cluster assembly in surface waters of the French‐side English Channel during the ECOPEL 2018 summer cruise (SIMPER results).
**Figure S4:**. Canonical correspondence analysis (CCA) of biogeochemical variables and bacterial taxa. Canonical correspondence analysis (CCA) biplots of bacterial taxa against environmental variables in surface waters of the French‐side English Channel in spring (top part) and summer (bottom part) during the ECOPEL 2018 cruises. The arrows represent the extent of the environmental variables, while the most distributed bacteria taxa are indicated in red. Chl‐*a*: Chlorophyll‐*a*; NO2 + NO3: nitrite + nitrate; Si: Silicate; DOC: dissolved organic carbon; SPM: suspended matter; Pheo: Phaeopigments.

**Table S1:** emi470213‐sup‐0002‐TableS1.xlsx.

## Data Availability

The data that support the findings of this study are openly available in the NCBI Sequence Read Archive at https://www.ncbi.nlm.nih.gov/sra, reference number PRJNA1242094.

## References

[emi470213-bib-0001] Alonso‐Sáez, L. , L. Díaz‐Pérez , and X. A. G. Morán . 2015. “The Hidden Seasonality of the Rare Biosphere in Coastal Marine Bacterioplankton.” Environmental Microbiology 17, no. 10: 3766–3780. 25684402 10.1111/1462-2920.12801

[emi470213-bib-0002] Altschul, S. F. , W. Gish , W. Miller , E. W. Myers , and D. J. Lipman . 1990. “Basic Local Alignment Search Tool.” Journal of Molecular Biology 215, no. 3: 403–410. 2231712 10.1016/S0022-2836(05)80360-2

[emi470213-bib-0003] Aminot, A. , R. Kerouel , and M.E.R. Institut Francais De Recherche Pour L'Exploitation De La . 2004. “Hydrologie des écosystèmes marins – paramètres et analyses.” Methodes D'analyse en Milieu Marin 29: 336.

[emi470213-bib-0004] Artigas, L. F. 2018. “ECOPEL 2018 Cruise, RV Antea.”

[emi470213-bib-0005] Azam, F. , T. Fenchel , J. G. Field , J. S. Gray , L. A. Meyer‐Reil , and F. Thingstad . 1983. “The Ecological Role of Water‐Column Microbes in the Sea.” Marine Ecology Progress Series 10: 257–263.

[emi470213-bib-0006] Barak‐Gavish, N. , M. J. F. Noa , C. Ku , et al. 2018. “Bacterial Virulence Against an Oceanic Bloom‐Forming Phytoplankter Is Mediated by Algal DMSP.” Science Advances 4, no. 10: eaau5716. 30397652 10.1126/sciadv.aau5716 PMC6200362

[emi470213-bib-0007] Behnke, A. , M. Engel , R. Christen , M. Nebel , R. R. Klein , and T. Stoeck . 2011. “Depicting More Accurate Pictures of Protistan Community Complexity Using Pyrosequencing of Hypervariable SSU rRNA Gene Regions.” Environmental Microbiology 13, no. 2: 340–349. 21281421 10.1111/j.1462-2920.2010.02332.x

[emi470213-bib-0008] Beiralas, R. , N. Ozer , and E. Segev . 2023. “Abundant Sulfitobacter Marine Bacteria Protect *Emiliania huxleyi* Algae From Pathogenic Bacteria.” ISME Communications 3, no. 1: 100. 37740057 10.1038/s43705-023-00311-y PMC10517135

[emi470213-bib-0009] Béjà, O. , L. Aravind , E. V. Koonin , et al. 2000. “Bacterial Rhodopsin: Evidence for a New Type of Phototrophy in the Sea.” Science 289, no. 5486: 1902–1906. 10988064 10.1126/science.289.5486.1902

[emi470213-bib-0010] Berger, W. H. , and F. L. Parker . 1970. “Diversity of Planktonic Foraminifera in Deep‐Sea Sediments.” Science 168, no. 3937: 1345–1347. 17731043 10.1126/science.168.3937.1345

[emi470213-bib-0011] Biessy, L. , J. K. Pearman , K. N. Mertens , et al. 2024. “Sudden Peak in Tetrodotoxin in French Oysters During the Summer of 2021: Source Investigation Using Microscopy, Metabarcoding and Droplet Digital PCR.” Toxicon 243: 107721. 38636612 10.1016/j.toxicon.2024.107721

[emi470213-bib-0012] Biessy, L. , J. K. Pearman , K. F. Smith , I. Hawes , and S. A. Wood . 2020. “Seasonal and Spatial Variations in Bacterial Communities From Tetrodotoxin‐Bearing and Non‐Tetrodotoxin‐Bearing Clams.” Frontiers in Microbiology 11: 1860. 32849450 10.3389/fmicb.2020.01860 PMC7419435

[emi470213-bib-0013] Bray, J. R. , and J. T. Curtis . 1957. “An Ordination of the Upland Forest Communities of Southern Wisconsin.” Ecological Monographs 27, no. 4: 325–349.

[emi470213-bib-0014] Brunet, C. , J. M. Brylinski , L. Bodineau , G. Thoumelin , D. Bentley , and D. Hilde . 1996. “Phytoplankton Dynamics During the Spring Bloom in the South‐Eastern English Channel.” Estuarine, Coastal and Shelf Science 43, no. 4: 469–483.

[emi470213-bib-0015] Brylinski, J. M. , Y. Lagadeuc , V. Gentilhomme , et al. 1991. “Le "fleuve côtier": un Phénomène Hydrologique Important en Manche Orientale. Exemple du Pas‐de‐Calais.” Oceallologica Acta, Proceedings of the International Colloquium on the Environment of Epicontinental Seas, March 1990. vol. sp.11, 197–203.

[emi470213-bib-0016] Buchan, A. , G. R. LeCleir , C. A. Gulvik , and J. M. González . 2014. “Master Recyclers: Features and Functions of Bacteria Associated With Phytoplankton Blooms.” Nature Reviews. Microbiology 12, no. 10: 686–698. 25134618 10.1038/nrmicro3326

[emi470213-bib-0017] Câmara Dos Reis, M. , S. Romac , F. le Gall , et al. 2023. “Exploring the Phycosphere of *Emiliania huxleyi* : From Bloom Dynamics to Microbiome Assembly Experiments.” Molecular Ecology 32, no. 23: 6507–6522. 36541038 10.1111/mec.16829

[emi470213-bib-0018] Caracciolo, M. , F. Rigaut‐Jalabert , S. Romac , et al. 2022. “Seasonal Dynamics of Marine Protist Communities in Tidally Mixed Coastal Waters.” Molecular Ecology 31, no. 14: 3761–3783. 35593305 10.1111/mec.16539 PMC9543310

[emi470213-bib-0019] Chao, A. 1984. “Nonparametric Estimation of the Number of Classes in a Population.” Scandinavian Journal of Statistics 11, no. 4: 265–270.

[emi470213-bib-0020] Charrad, M. , N. Ghazzali , V. Boiteau , and A. Niknafs . 2014. “NbClust: An R Package for Determining the Relevant Number of Clusters in a Data Set.” Journal of Statistical Software 61, no. 6: 1–36.

[emi470213-bib-0021] Clarke, K. R. , and R. N. Gorley . 2006. PRIMER v6: User Manual/Tutorial. By PRIMER‐E Ltd, Plymouth Marine Laboratory, Plymouth, UK.

[emi470213-bib-0022] Cooley, S. , D. Schoeman , L. Bopp , et al. 2022. “Chapter 3: Oceans and Coastal Ecosystems and Their Services, in Climate Change 2022: Impacts, Adaptation and Vulnerability.” In IPCC WGII Sixth Assessment Report. By The Intergovernmental Panel on Climate Change, United Nations.

[emi470213-bib-0023] Dauvin, J. C. 2012. “Are the Eastern and Western Basins of the English Channel Two Separate Ecosystems?” Marine Pollution Bulletin 64, no. 3: 463–471. 22245434 10.1016/j.marpolbul.2011.12.010

[emi470213-bib-0024] du Bois, P. B. , and F. Dumas . 2005. “Fast Hydrodynamic Model for Medium‐ and Long‐Term Dispersion in Seawater in the English Channel and Southern North Sea, Qualitative and Quantitative Validation by Radionuclide Tracers.” Ocean Modelling 9, no. 2: 169–210.

[emi470213-bib-0025] Dunn, J. C. 1974. “Well‐Separated Clusters and Optimal Fuzzy Partitions.” Journal of Cybernetics 4, no. 1: 95–104.

[emi470213-bib-0026] Edgar, R. C. 2010. “Search and Clustering Orders of Magnitude Faster Than BLAST.” Bioinformatics 26, no. 19: 2460–2461. 20709691 10.1093/bioinformatics/btq461

[emi470213-bib-0027] Fandino, L. B. , L. Riemann , G. F. Steward , R. A. Long , and F. Azam . 2001. “Variations in Bacterial Community Structure During a Dinoflagellate Bloom Analyzed by DGGE and 16S rDNA Sequencing.” Aquatic Microbial Ecology 23: 119–130.

[emi470213-bib-0028] Freitas, S. , S. Hatosy , J. A. Fuhrman , et al. 2012. “Global Distribution and Diversity of Marine Verrucomicrobia.” ISME Journal 6, no. 8: 1499–1505. 22318305 10.1038/ismej.2012.3 PMC3400412

[emi470213-bib-0029] Ghai, R. , C. M. Mizuno , A. Picazo , A. Camacho , and F. Rodriguez‐Valera . 2013. “Metagenomics Uncovers a New Group of Low GC and Ultra‐Small Marine Actinobacteria.” Scientific Reports 3: 2471. 23959135 10.1038/srep02471 PMC3747508

[emi470213-bib-0030] Giebel, H.‐A. , M. Wolterink , T. Brinkhoff , and M. Simon . 2019. “Complementary Energy Acquisition via Aerobic Anoxygenic Photosynthesis and Carbon Monoxide Oxidation by Planktomarina Temperata of the Roseobacter Group.” FEMS Microbiology Ecology 95, no. 5: fiz050. 31055603 10.1093/femsec/fiz050

[emi470213-bib-0031] Gilbert, J. A. , D. Field , P. Swift , et al. 2009. “The Seasonal Structure of Microbial Communities in the Western English Channel.” Environmental Microbiology 11, no. 12: 3132–3139. 19659500 10.1111/j.1462-2920.2009.02017.x

[emi470213-bib-0032] Gilbert, J. A. , J. A. Steele , J. G. Caporaso , et al. 2012. “Defining Seasonal Marine Microbial Community Dynamics.” ISME Journal 6, no. 2: 298–308. 21850055 10.1038/ismej.2011.107 PMC3260500

[emi470213-bib-0033] Giovannoni, S. J. 2017. “SAR11 Bacteria: The Most Abundant Plankton in the Oceans.” Annual Review of Marine Science 9: 231–255. 10.1146/annurev-marine-010814-015934 27687974

[emi470213-bib-0034] Glegg, G. , R. Jefferson , and S. Fletcher . 2015. “Marine Governance in the English Channel (La Manche): Linking Science and Management.” Marine Pollution Bulletin 95, no. 2: 707–718. 25819447 10.1016/j.marpolbul.2015.02.020

[emi470213-bib-0035] Goberville, E. , G. Beaugrand , B. Sautour , P. Tréguer , and SOMLIT Team . 2010. “Climate‐Driven Changes in Coastal Marine Systems of Western Europe.” Marine Ecology Progress Series 408: 129–148.

[emi470213-bib-0036] Gómez‐Consarnau, L. , D. M. Needham , P. K. Weber , J. A. Fuhrman , and X. Mayali . 2019. “Influence of Light on Particulate Organic Matter Utilization by Attached and Free‐Living Marine Bacteria.” Frontiers in Microbiology 10: 1204. 31214143 10.3389/fmicb.2019.01204 PMC6558058

[emi470213-bib-0037] Goodwin, K. D. , L. R. Thompson , B. Duarte , et al. 2017. “DNA Sequencing as a Tool to Monitor Marine Ecological Status.” Frontiers in Marine Science 4: 107.

[emi470213-bib-0038] Grieb, A. , T. B. Francis , K. Krüger , L. H. Orellana , R. Amann , and B. M. Fuchs . 2020. “Candidatus Abditibacter, a Novel Genus Within the Cryomorphaceae, Thriving in the North Sea.” Systematic and Applied Microbiology 43, no. 4: 126088. 32690198 10.1016/j.syapm.2020.126088

[emi470213-bib-0039] Group, G . 1988. “A Physical, Chemical and Biological Characterization of the Ushant Tidal Front.” Internationale Revue der Gesamten Hydrobiologie und Hydrographie 73, no. 5: 511–536.

[emi470213-bib-0040] Hahnke, R. L. , C. M. Bennke , B. M. Fuchs , et al. 2015. “Dilution Cultivation of Marine Heterotrophic Bacteria Abundant After a Spring Phytoplankton Bloom in the North Sea.” Environmental Microbiology 17, no. 10: 3515–3526. 24725270 10.1111/1462-2920.12479

[emi470213-bib-0041] Hammer, O. , D. Harper , and P. Ryan . 2001. “PAST: Paleontological Statistics Software Package for Education and Data Analysis.” Palaeontologia Electronica 4: 1–9.

[emi470213-bib-0042] Han, M. , M. Dsouza , C. Zhou , et al. 2019. “Agricultural Risk Factors Influence Microbial Ecology in Honghu Lake.” Genomics, Proteomics & Bioinformatics 17, no. 1: 76–90. 10.1016/j.gpb.2018.04.008 PMC6520916 31026580

[emi470213-bib-0043] Hoch, T. , and A. Ménesguen . 1997. “Modelling the Biogeochemical Cycles of Elements Limiting Primary Production in the English Channel. II. Sensitivity Analyses.” Marine Ecology Progress Series 146: 189–205.

[emi470213-bib-0044] Idier, D. , F. Dumas , and H. Muller . 2012. “Tide‐Surge Interaction in the English Channel.” Natural Hazards and Earth System Sciences 12, no. 12: 3709–3718.

[emi470213-bib-0045] James, R. 2005. “Marine Biogeochemical Cycles.” In Elsevier Oceanography Series, Ed. T. Open University. Oceanography Course. Open University.

[emi470213-bib-0046] Klindworth, A. , E. Pruesse , T. Schweer , et al. 2013. “Evaluation of General 16S Ribosomal RNA Gene PCR Primers for Classical and Next‐Generation Sequencing‐Based Diversity Studies.” Nucleic Acids Research 41, no. 1: e1. 22933715 10.1093/nar/gks808 PMC3592464

[emi470213-bib-0047] Kozich, J. J. , S. L. Westcott , N. T. Baxter , S. K. Highlander , and P. D. Schloss . 2013. “Development of a Dual‐Index Sequencing Strategy and Curation Pipeline for Analyzing Amplicon Sequence Data on the MiSeq Illumina Sequencing Platform.” Applied and Environmental Microbiology 79, no. 17: 5112–5120. 23793624 10.1128/AEM.01043-13 PMC3753973

[emi470213-bib-0048] Kunin, V. , A. Engelbrektson , H. Ochman , and P. Hugenholtz . 2010. “Wrinkles in the Rare Biosphere: Pyrosequencing Errors Can Lead to Artificial Inflation of Diversity Estimates.” Environmental Microbiology 12, no. 1: 118–123. 19725865 10.1111/j.1462-2920.2009.02051.x

[emi470213-bib-0049] Lamy, D. , I. Obernosterer , M. Laghdass , et al. 2009. “Temporal Changes of Major Bacterial Groups and Bacterial Heterotrophic Activity During a *Phaeocystis globosa* Bloom in the Eastern English Channel.” Aquatic Microbial Ecology 58, no. 1: 95–107.

[emi470213-bib-0050] Lê, S. , J. Josse , and F. Husson . 2008. “FactoMineR: An R Package for Multivariate Analysis.” Journal of Statistical Software 25, no. 1: 1–18.

[emi470213-bib-0051] Lee, J. , K. K. Kwon , S. I. Lim , et al. 2019. “Isolation, Cultivation, and Genome Analysis of Proteorhodopsin‐Containing SAR116‐Clade Strain Candidatus Puniceispirillum Marinum IMCC1322.” Journal of Microbiology 57, no. 8: 676–687. 31201724 10.1007/s12275-019-9001-2

[emi470213-bib-0052] Lefebvre, A. , and C. Dezécache . 2020. “Trajectories of Changes in Phytoplankton Biomass, Phaeocystis Globosa and Diatom (Incl. Pseudo‐Nitzschia sp.) Abundances Related to Nutrient Pressures in the Eastern English Channel, Southern North Sea.” Journal of Marine Science and Engineering 8, no. 6: 401.

[emi470213-bib-0053] Lemonnier, C. , M. Perennou , D. Eveillard , et al. 2020. “Linking Spatial and Temporal Dynamic of Bacterioplankton Communities With Ecological Strategies Across a Coastal Frontal Area.” Frontiers in Marine Science 7: 376.

[emi470213-bib-0054] Lloyd, M. , and R. J. Ghelardi . 1964. “A Table for Calculating the ‘Equitability’ Component of Species Diversity.” Journal of Animal Ecology 33, no. 2: 217–225.

[emi470213-bib-0055] López‐García, P. , and D. Moreira . 2008. “Tracking Microbial Biodiversity Through Molecular and Genomic Ecology.” Research in Microbiology 159, no. 1: 67–73. 18207371 10.1016/j.resmic.2007.11.019

[emi470213-bib-0056] Mantel, N. 1967. “The Detection of Disease Clustering and a Generalized Regression Approach.” Cancer Research 27, no. 2: 209–220. 6018555

[emi470213-bib-0057] Marie, D. , X. L. Shi , F. Rigaut‐Jalabert , and D. Vaulot . 2010. “Use of Flow Cytometric Sorting to Better Assess the Diversity of Small Photosynthetic Eukaryotes in the English Channel.” FEMS Microbiology Ecology 72, no. 2: 165–178. 20236325 10.1111/j.1574-6941.2010.00842.x

[emi470213-bib-0058] Moreira, D. , and P. López‐García . 2002. “The Molecular Ecology of Microbial Eukaryotes Unveils a Hidden World.” Trends in Microbiology 10, no. 1: 31–38. 11755083 10.1016/s0966-842x(01)02257-0

[emi470213-bib-0059] Murtagh, F. , and P. Legendre . 2014. “Ward's Hierarchical Agglomerative Clustering Method: Which Algorithms Implement Ward's Criterion?” Journal of Classification 31, no. 3: 274–295.

[emi470213-bib-0060] Neteler, M. , M. H. Bowman , M. Landa , and M. Metz . 2012. “GRASS GIS: A Multi‐Purpose Open Source GIS.” Environmental Modelling and Software 31: 124–130.

[emi470213-bib-0061] Normand, P. , R. Duran , X. Le Roux , et al. 2015. “Biodiversity and Microbial Ecosystems Functioning.” In Environmental Microbiology: Fundamentals and Applications, 261–291. Springer.

[emi470213-bib-0062] Oksanen, J. , F. G. Blanchet , R. Kindt , et al. 2020. “Vegan Community Ecology Package Version 2.5–7.”

[emi470213-bib-0063] Pielou, E. C. 1966. “The Measurement of Diversity in Different Types of Biological Collections.” Journal of Theoretical Biology 13: 131–144.

[emi470213-bib-0064] Pingree, R. D. , and D. K. Griffiths . 1978. “Tidal Fronts on the Shelf Seas Around the British Isles.” Journal of Geophysical Research: Oceans 83, no. C9: 4615–4622.

[emi470213-bib-0065] Pinhassi, J. , M. M. Sala , H. Havskum , et al. 2004. “Changes in Bacterioplankton Composition Under Different Phytoplankton Regimens.” Applied and Environmental Microbiology 70, no. 11: 6753–6766. 15528542 10.1128/AEM.70.11.6753-6766.2004 PMC525254

[emi470213-bib-0066] Quast, C. , E. Pruesse , P. Yilmaz , et al. 2013. “The SILVA Ribosomal RNA Gene Database Project: Improved Data Processing and Web‐Based Tools.” Nucleic Acids Research 41: D590–D596. 23193283 10.1093/nar/gks1219 PMC3531112

[emi470213-bib-0067] R Core Team . 2021. “R: A Language and Environment for Statistical Computing.” In By R Foundation for Statistical Computing.

[emi470213-bib-0068] Rachik, S. , U. Christaki , L. L. Li , S. Genitsaris , E. Breton , and S. Monchy . 2018. “Diversity and Potential Activity Patterns of Planktonic Eukaryotic Microbes in a Mesoeutrophic Coastal Area (Eastern English Channel).” PLoS One 13, no. 5: e0196987. 29746519 10.1371/journal.pone.0196987 PMC5944946

[emi470213-bib-0069] Reynaud, J.‐Y. , B. Tessier , J. P. Auffret , et al. 2003. “The Offshore Quaternary Sediment Bodies of the English Channel and Its Western Approaches.” Journal of Quaternary Science 18, no. 3–4: 361–371.

[emi470213-bib-0070] Riemann, L. , G. F. Steward , and F. Azam . 2000. “Dynamics of Bacterial Community Composition and Activity During a Mesocosm Diatom Bloom.” Applied and Environmental Microbiology 66, no. 2: 578–587. 10653721 10.1128/aem.66.2.578-587.2000 PMC91866

[emi470213-bib-0071] Rink, B. , S. Seeberger , T. Martens , D. Duerselen C , M. Simon , and T. Brinkhoff . 2007. “Effects of Phytoplankton Bloom in a Coastal Ecosystem on the Composition of Bacterial Communities.” Aquatic Microbial Ecology 48, no. 1: 47–60.

[emi470213-bib-0072] Rodríguez, J. , C. M. J. Gallampois , S. Timonen , et al. 2018. “Effects of Organic Pollutants on Bacterial Communities Under Future Climate Change Scenarios.” Frontiers in Microbiology 9: 2926. 30555447 10.3389/fmicb.2018.02926 PMC6284067

[emi470213-bib-0073] Salomon, J.‐C. , and M. Breton . 1993. “An Atlas of Long‐Term Currents in the Channel.” Oceanologica Acta 16, no. 5–6: 439–448.

[emi470213-bib-0074] Schloss, P. D. , S. L. Westcott , T. Ryabin , et al. 2009. “Introducing Mothur: Open‐Source, Platform‐Independent, Community‐Supported Software for Describing and Comparing Microbial Communities.” Applied and Environmental Microbiology 75, no. 23: 7537–7541. 19801464 10.1128/AEM.01541-09 PMC2786419

[emi470213-bib-0075] Seyedsayamdost, M. R. , R. J. Case , R. Kolter , and J. Clardy . 2011. “The Jekyll‐and‐Hyde Chemistry of *Phaeobacter gallaeciensis* .” Nature Chemistry 3, no. 4: 331–335. 10.1038/nchem.1002 PMC3376411 21430694

[emi470213-bib-0076] Shannon, C. E. 1948. “A Mathematical Theory of Communication.” Bell System Technical Journal 27, no. 3: 379–423.

[emi470213-bib-0077] Sichert, A. , C. H. Corzett , M. S. Schechter , et al. 2020. “Verrucomicrobia Use Hundreds of Enzymes to Digest the Algal Polysaccharide Fucoidan.” Nature Microbiology 5, no. 8: 1026–1039. 10.1038/s41564-020-0720-2 32451471

[emi470213-bib-0078] Simpson, E. H. 1949. “Measurement of Diversity.” Nature 163, no. 4148: 688.

[emi470213-bib-0079] Skouroliakou, D.‐I. , E. Breton , and U. Christaki . 2024. “Phaeocystis Globosa and Diatom Blooms Promote Distinct Bacterial Communities and Associations in a Coastal Ecosystem.” Environmental Microbiology Reports 16, no. 4: e13313. 38988030 10.1111/1758-2229.13313 PMC11236930

[emi470213-bib-0080] Southward, A. J. , O. Langmead , N. J. Hardman‐Mountford , et al. 2005. “Long‐Term Oceanographic and Ecological Research in the Western English Channel.” Advances in Marine Biology 47: 1–105. 15596166 10.1016/S0065-2881(04)47001-1

[emi470213-bib-0081] Spilmont, N. , L. Denis , L. F. Artigas , et al. 2009. “Impact of the *Phaeocystis globosa* Spring Bloom on the Intertidal Benthic Compartment in the Eastern English Channel: A Synthesis.” Marine Pollution Bulletin 58, no. 1: 55–63. 18947841 10.1016/j.marpolbul.2008.09.007

[emi470213-bib-0082] Suberg, L. A. , P. I. Miller , and R. B. Wynn . 2019. “On the Use of Satellite‐Derived Frontal Metrics in Time Series Analyses of Shelf‐Sea Fronts, a Study of the Celtic Sea.” Deep Sea Research Part I: Oceanographic Research Papers 149: 103033.

[emi470213-bib-0083] Tarran, G. A. , and J. T. Bruun . 2015. “Nanoplankton and Picoplankton in the Western English Channel: Abundance and Seasonality From 2007–2013.” Progress in Oceanography 137: 446–455.

[emi470213-bib-0084] Teeling, H. , B. M. Fuchs , D. Becher , et al. 2012. “Substrate‐Controlled Succession of Marine Bacterioplankton Populations Induced by a Phytoplankton Bloom.” Science 336, no. 6081: 608–611. 22556258 10.1126/science.1218344

[emi470213-bib-0085] ter Braak, C. J. F. 1986. “Canonical Correspondence Analysis: A New Eigenvector Technique for Multivariate Direct Gradient Analysis.” Ecology 67, no. 5: 1167–1179.

[emi470213-bib-0086] Tripp, H. J. , J. B. Kitner , M. S. Schwalbach , J. W. H. Dacey , L. J. Wilhelm , and S. J. Giovannoni . 2008. “SAR11 Marine Bacteria Require Exogenous Reduced Sulphur for Growth.” Nature 452, no. 7188: 741–744. 18337719 10.1038/nature06776

[emi470213-bib-0087] Videau, C. , M. Ryckaert , and S. L'Helguen . 1998. “Phytoplancton en baie de Seine. Influence du panache fluvial sur la production primaire.” Oceanologica Acta 21, no. 6: 907–921.

[emi470213-bib-0088] von Scheibner, M. , U. Sommer , and K. Jürgens . 2017. “Tight Coupling of Glaciecola spp. and Diatoms During Cold‐Water Phytoplankton Spring Blooms.” Frontiers in Microbiology 8: 27. 28154558 10.3389/fmicb.2017.00027 PMC5243806

[emi470213-bib-0089] Ward, J. H., Jr. 1963. “Hierarchical Grouping to Optimize an Objective Function.” Journal of the American Statistical Association 58, no. 301: 236–244.

[emi470213-bib-0090] Wemheuer, B. , F. Wemheuer , J. Hollensteiner , et al. 2015. “The Green Impact: Bacterioplankton Response Toward a Phytoplankton Spring Bloom in the Southern North Sea Assessed by Comparative Metagenomic and Metatranscriptomic Approaches.” Frontiers in Microbiology 6: 805. 26322028 10.3389/fmicb.2015.00805 PMC4531512

[emi470213-bib-0091] Widdicombe, C. E. , D. Eloire , D. Harbour , R. P. Harris , and P. J. Somerfield . 2010. “Long‐Term Phytoplankton Community Dynamics in the Western English Channel.” Journal of Plankton Research 32, no. 5: 643–655.

[emi470213-bib-0092] World Meteorological Organization, U.N . 2023. The Global Climate 2011–2020: A Decade of Accelerating Climate Change. United Nations.

[emi470213-bib-0093] Xu, X. , M. He , Q. Xue , X. Li , and A. Liu . 2024. “Genome‐Based Taxonomic Classification of the Genus Sulfitobacter Along With the Proposal of a New Genus Parasulfitobacter Gen. Nov. and Exploring the Gene Clusters Associated With Sulfur Oxidation.” BMC Genomics 25, no. 1: 389. 38649849 10.1186/s12864-024-10269-3 PMC11034169

[emi470213-bib-0094] Yoch, D. C. 2002. “Dimethylsulfoniopropionate: Its Sources, Role in the Marine Food Web, and Biological Degradation to Dimethylsulfide.” Applied and Environmental Microbiology 68, no. 12: 5804–5815. 12450799 10.1128/AEM.68.12.5804-5815.2002 PMC134419

[emi470213-bib-0095] Zhang, Y. S. , Y. Q. Zhang , X. M. Zhao , et al. 2024. “Metagenomic Insights Into the Dynamic Degradation of Brown Algal Polysaccharides by Kelp‐Associated Microbiota.” Applied and Environmental Microbiology 90, no. 2: e0202523. 38259074 10.1128/aem.02025-23 PMC10880675

