# Supplementary material for: Environmental Variables Influence on Spatial Distribution of Bacterial Communities Across the English Channel in Two Main Productive Seasons
Source: Environ Microbiol Rep. 2025 Oct 12;17(5):e70213. doi: 10.1111/1758-2229.70213 (PMC12515505; doi:10.1111/1758-2229.70213)
Supplement: Supplementary file 1 — Figure S1: Map of the study area during the ECOPEL 2018 cruises. Figure S2: Taxonomic composition at each station in surface waters of the French‐side English Channel during the ECOPEL cruises. ‘Others’ represents all phyla that have their highest proportion lower than 1%. Figure S3:. Heatmaps of the OTUs identified by SIMPER analysis as contributing the most (> 1%) to the clusters observed in (a) spring and (b) summer. The contributions are indicated with a colour gradient (see scale on the right side of the figure) with light yellow corresponding to low percentage of contribution and red to high contribution. (a) Bacterial contribution of each cluster assembly in surface waters of the French‐side English Channel during the ECOPEL 2018 spring cruise (SIMPER results). (b) Bacterial contribution of each cluster assembly in surface waters of the French‐side English Channel during the ECOPEL 2018 summer cruise (SIMPER results). Figure S4:. Canonical correspondence analysis (CCA) of biogeochemical variables and bacterial taxa. Canonical correspondence analysis (CCA) biplots of bacterial taxa against environmental variables in surface waters of the French‐side English Channel in spring (top part) and summer (bottom part) during the ECOPEL 2018 cruises. The arrows represent the extent of the environmental variables, while the most distributed bacteria taxa are indicated in red. Chl‐a: Chlorophyll‐a; NO2 + NO3: nitrite + nitrate; Si: Silicate; DOC: dissolved organic carbon; SPM: suspended matter; Pheo: Phaeopigments. [file EMI4-17-e70213-s002.pdf]

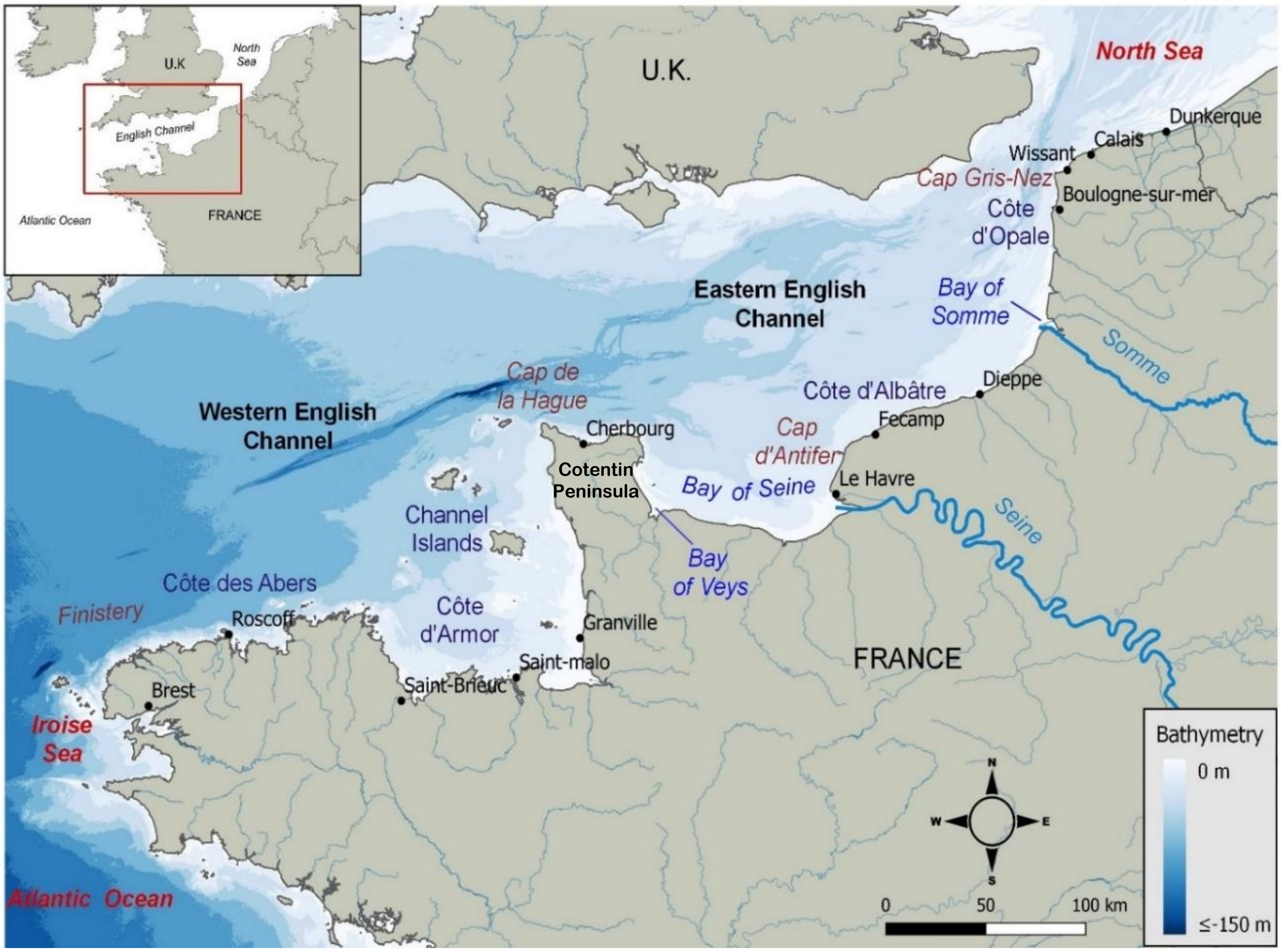

Supporting Information Fig. 1. Map of the study area during the ECOPEL 2018 cruises

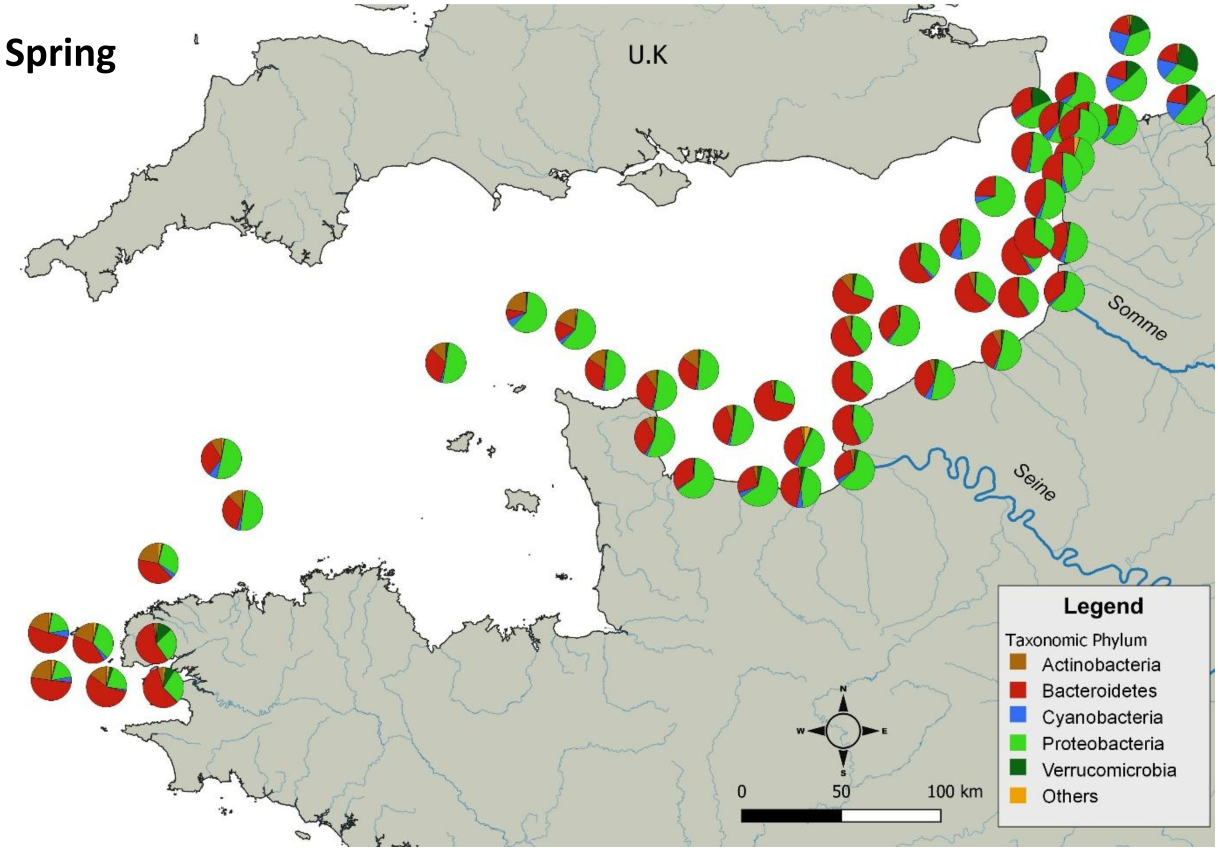

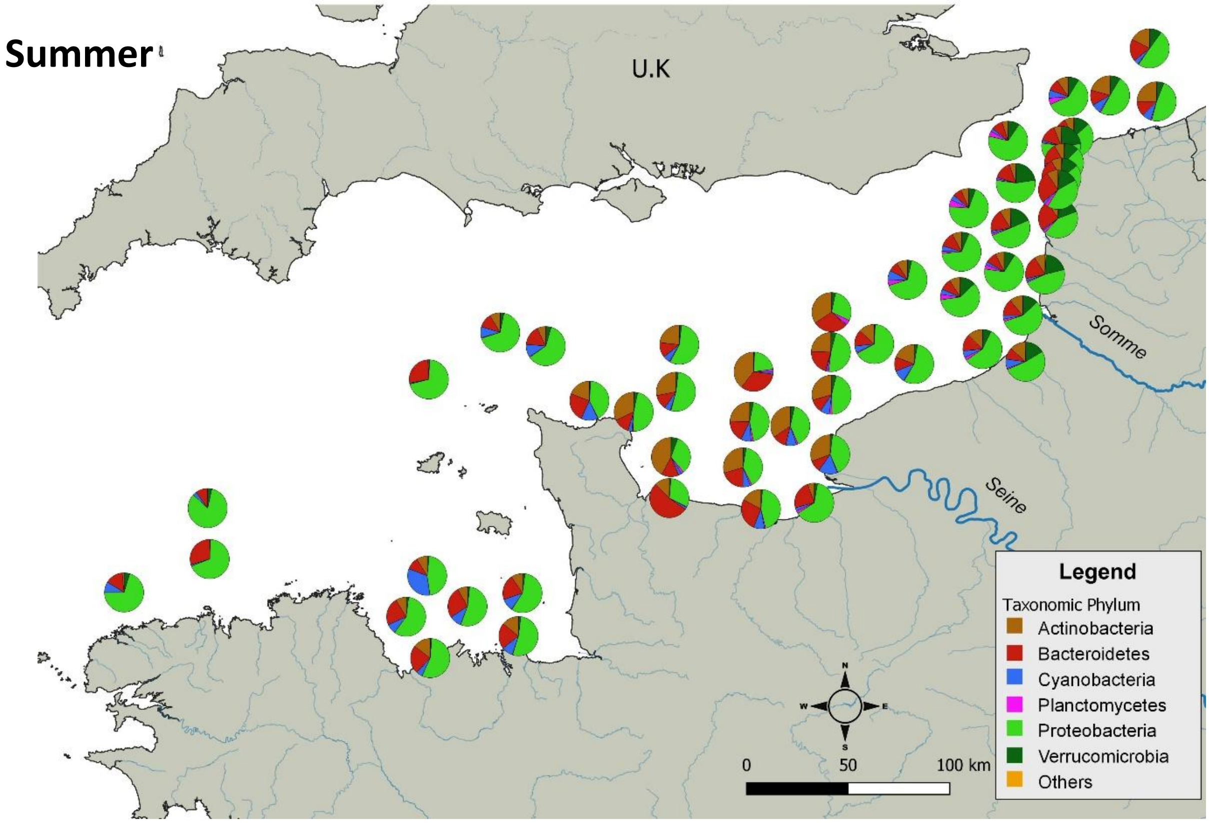

Supporting Information Fig. 2. Taxonomic composition at each station in surface waters of the French-side English Channel during the ECOPEL cruises. "Others" represents all phyla that have their highest proportion lower than 1%.

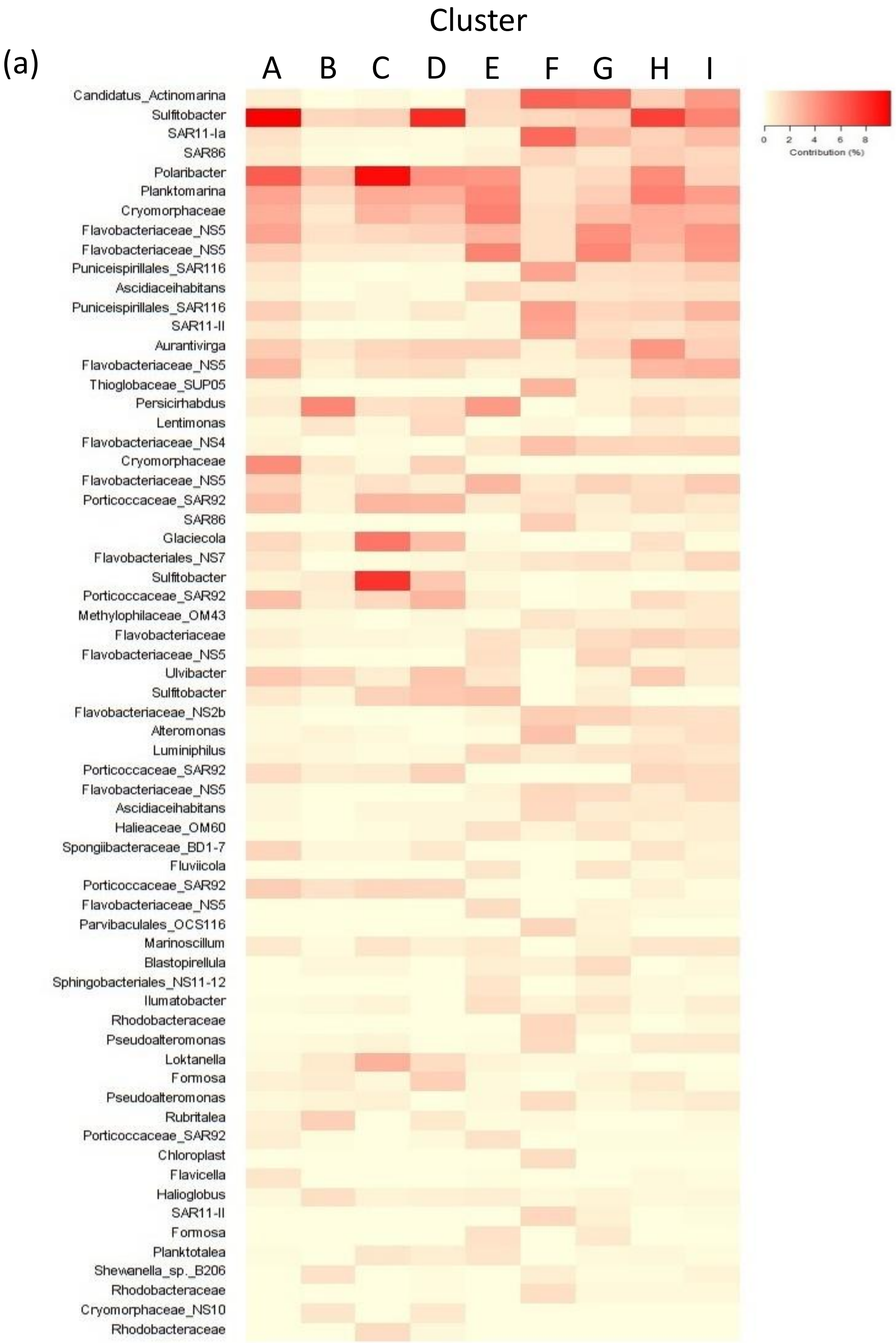

Supporting Information Fig. 3a. Bacterial contribution of each cluster assembly in surface waters of the French-side English Channel during the ECOPEL 2018 spring cruise (SIMPER results)

(b)

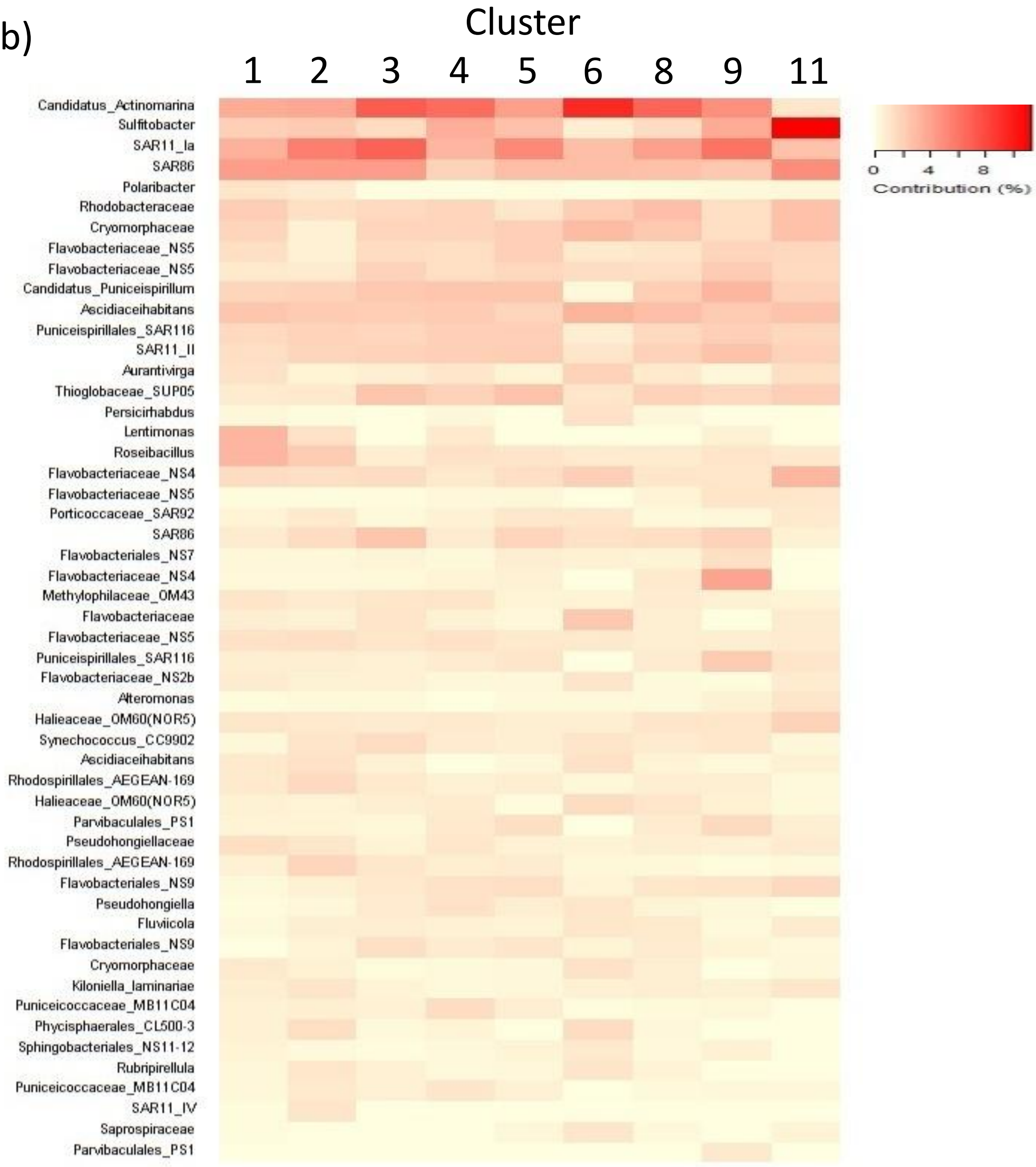

Supporting Information Fig. 3b. Bacterial contribution of each cluster assembly in surface waters of the French-side English Channel during the ECOPEL 2018 summer cruise (SIMPER results)

Supporting Information Fig. 3.

Heatmaps of the OTUs identified by SIMPER analysis as contributing the most (>1%) to the clusters observed in (a) spring and (b) summer. The contributions are indicated with a color gradient (see scale on the right side of the figure) with light yellow corresponding to low percentage of contribution and red to high contribution.

Spring

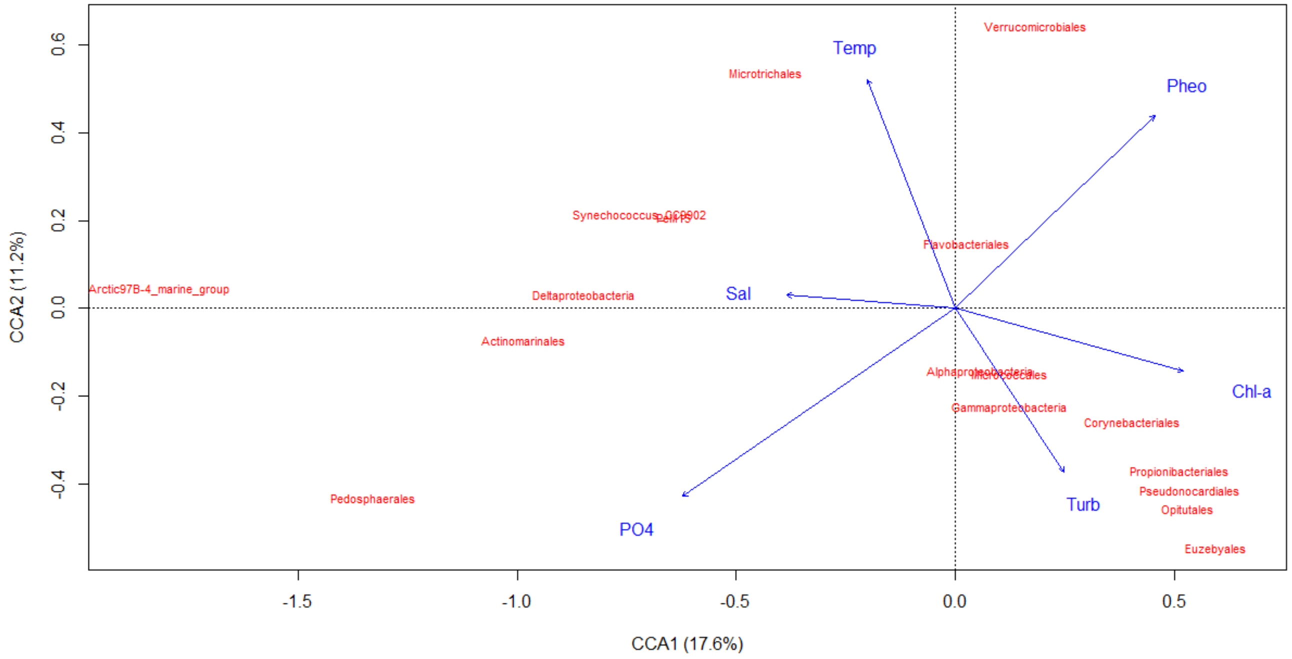

Summer

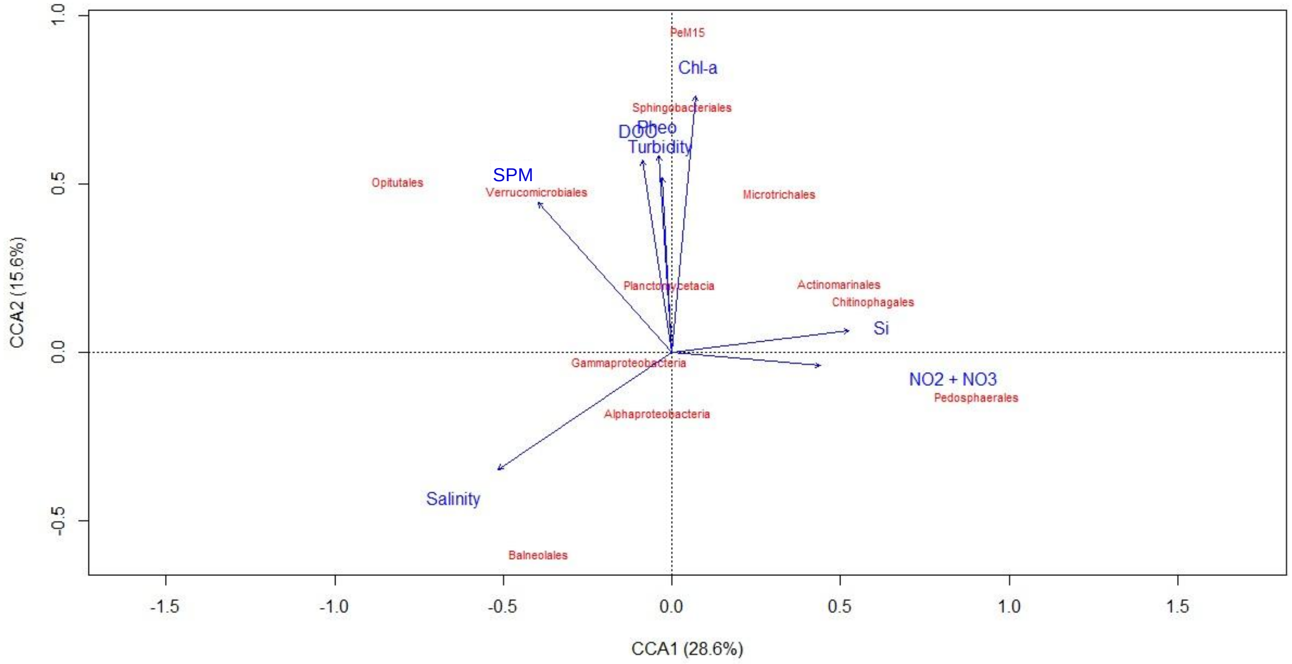

Supporting Information Fig. 4. Canonical correspondence analysis (CCA) of biogeochemical variables and bacterial taxa

Canonical correspondence analysis (CCA) biplots of bacterial taxa against environmental variables in surface waters of the French-side English Channel in spring (top part) and summer (bottom part) during the ECOPEL 2018 cruises. The arrows represent the extent of the environmental variables, while the most distributed bacteria taxa are indicated in red. Chl-*a*: Chlorophyll-*a;* NO_2+NO_3: nitrite + nitrate; Si: Silicate; DOC: dissolved organic carbon; SPM: suspended matter; Pheo: Phaeopigments
